# The Role of Conditional Likelihoods in Latent Variable Modeling

**DOI:** 10.1007/s11336-021-09816-8

**Published:** 2022-01-10

**Authors:** Anders Skrondal, Sophia Rabe-Hesketh

**Affiliations:** 1 CEFH, Norwegian Institute of Public Health, P.O.Box 222 Skøyen, N-0213 Oslo, Norway; 2 CEMO, University of Oslo, Oslo, Norway; 3 GSE, University of California, Berkeley, Berkeley, USA

**Keywords:** Endogeneity, Fixed effects, Random effects, Conditional maximum likelihood, Marginal maximum likelihood, Unobserved confounding, Measurement error, Retrospective sampling, Informative cluster size, Missing data, Heteroskedasticity

## Abstract

In psychometrics, the canonical use of conditional likelihoods is for the Rasch model in measurement. Whilst not disputing the utility of conditional likelihoods in measurement, we examine a broader class of problems in psychometrics that can be addressed via conditional likelihoods. Specifically, we consider cluster-level endogeneity where the standard assumption that observed explanatory variables are independent from latent variables is violated. Here, “cluster” refers to the entity characterized by latent variables or random effects, such as individuals in measurement models or schools in multilevel models and “unit” refers to the elementary entity such as an item in measurement. Cluster-level endogeneity problems can arise in a number of settings, including unobserved confounding of causal effects, measurement error, retrospective sampling, informative cluster sizes, missing data, and heteroskedasticity. Severely inconsistent estimation can result if these challenges are ignored.

## Introduction

As is often the case for concepts in statistics, the term “conditional likelihood” has many meanings. It has, for instance, been used to refer to likelihoods where conditioning is on (1) exogenous explanatory variables (e.g., Gourieroux & Monfort, 1995), (2) latent variables (e.g., Aigner et al., 1984), (3) the outcome variable, for instance in capture-recapture modeling of population size (e.g., Sanathanan, 1972) and ascertainment correction in biometrical genetics (e.g., Pfeiffer et al., 2001), (4) previous outcomes, for instance in autoregressive time-series models (e.g., Box & Jenkins, 1976) and peeling in phylogenetics (e.g., Felsenstein, 1981), (5) order statistics (e.g., Kalbfleisch, 1978), or (6) sufficient statistics.

In this address we follow the seminal theoretical work of Andersen (1970, 1973a) and Kalbfleisch and Sprott (1970) and let a conditional likelihood be obtained by *conditioning on sufficient statistics for incidental parameters in order to eliminate these parameters*. In the context of latent variable or mixed effects modeling, the incidental parameters are the values taken by latent variables for a set of clusters, for example individuals or organizational units.

In psychometrics, the canonical use of conditional likelihoods is in measurement relying on the Rasch model (Rasch, 1960) and its extensions. As demonstrated by Rasch, estimation of item parameters can in this case be based on a conditional likelihood where the person parameters are eliminated by conditioning on their sufficient statistics. It is often argued that Rasch models and conditional maximum likelihood (CML) estimation are advantageous in measurement (e.g., Fischer, 1995a). Indeed, Molenaar (1995) closes his excellent overview of estimation of Rasch models in the following way:

“Unless there are clear reasons for a different decision, the present author would recommend to use CML estimates.”

Conditional likelihoods have been used for a variety of problems in measurement; see Fischer (1995b; c), Formann (1995), Maris and Bechger (2007), Verhelst (2019), von Davier and Rost (1995), and Zwitser and Maris (2015) for a small selected sample.

We are certainly not disputing the utility of CML estimation in measurement. However, we will argue that conditional likelihoods perhaps have a more important role to play in addressing endogeneity problems in psychometrics. Focus will be on cluster-level endogeneity, where covariates and latent variables are dependent, a problem ignored by popular methods which can therefore produce severely inconsistent estimates. Fortunately, CML estimation, an instance of what is referred to as “fixed-effects estimation” in econometrics, can rectify this problem.

Our plan is as follows. First we introduce some latent variable models and discuss the cluster-level endogeneity problem whose origins, effects and alleviation we will examine. We proceed to delineate the ideas of protective and mitigating estimation of target parameters before describing the incidental parameter problem of joint maximum likelihood (JML) estimation. Two approaches that address that problem are discussed: marginal maximum likelihood (MML) and conditional maximum likelihood (CML) estimation. We demonstrate that CML estimation, in contrast to MML estimation, handles cluster-level endogeneity, and describe an endogeneity-correcting feature of MML estimation for large clusters. The scope of CML estimation is then extended followed by a discussion of MML estimation of augmented models that can accommodate cluster-level endogeneity. Several reasons for cluster-level endogeneity are investigated (unobserved cluster-level confounding of causal effects, cluster-specific measurement error, retrospective sampling, informative cluster sizes, missing data, and heteroskedasticity) and we show how different estimators perform in these situations. Thereafter, we discuss latent variable scoring before closing the paper with some concluding remarks.

## Clustered Data

We consider data consisting of clusters *j* ($j=1,\ldots ,N$) that contain units *ij* ($i=1,\ldots ,n_{j}$). Units are typically exchangeable within clusters in cross-sectional multilevel designs. An example is students *ij* nested in schools *j*, where the index *i* associated with the students within a school is arbitrary.

**Fig. 1:**
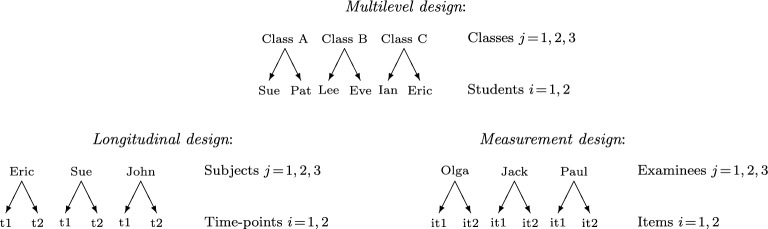
Illustration of clustered data for $N=3$ clusters and $n=2$ units per cluster. Exchangeable units (upper panel) and non-exchangeable units (lower panel).

Units are non-exchangeable within clusters in two settings: (a) longitudinal designs where *i* is the chronological sequence number of the time-point when a subject was observed and (b) measurement designs where *i* is the item (or question) responded to by a subject. In the non-exchangeable case, when *i* corresponds to the same time-point or item across subjects *j*, we will refer to *i* as an “item.” The different kinds of clustered data are illustrated in Fig. 1.

## Latent Variable Model

We consider generalized linear mixed models (GLMMs) with canonical link functions (see, e.g., Rabe-Hesketh & Skrondal, 2009). Given the cluster-specific latent variables or random effects ${\varvec{\zeta }}_{j}$, the model for an outcome $y_{ij}$ is a generalized linear model (GLM) with three components (e.g., Nelder & Wedderburn, 1972): a linear predictor $\nu _{ij}$, a link function $g(\mu _{ij})=\nu _{ij}$ that links the linear predictor to the conditional expectation $\mu _{ij}$ of the outcome, and a conditional outcome distribution from the exponential family.

For a GLMM, we express the linear predictor as

1 $$\begin{aligned} \nu _{ij} \ = \ {\mathbf {x}}_{ij}^{\prime }{\varvec{\beta }}+ {{\mathbf {v}}}_{j}^{\prime }{\varvec{\gamma }}+ {{\mathbf {z}}}_{ij}^{\prime }{\varvec{\zeta }}_{j}, \end{aligned}$$

where:

- ${\varvec{\beta }}$ is a vector of parameters for the unit-specific vector ${\mathbf {x}}_{ij}$. For exchangeable units ${\varvec{\beta }}$ are regression coefficients for unit-specific covariates ${\mathbf {x}}_{ij}$. For non-exchangeable units ${\varvec{\beta }}$ contains a vector of item-specific intercepts and a vector of regression coefficients. Correspondingly, ${\mathbf {x}}_{ij}$ includes an elementary vector (where one of the elements is 1 and the other elements are 0) that picks out the intercept for item *i*, and item-specific and/or unit-specific covariates.
- ${\varvec{\gamma }}$ is a vector of parameters for the cluster-specific vector ${{\mathbf {v}}}_{j}$. For non-exchangeable units ${\varvec{\gamma }}$ are regression coefficients for cluster-specific covariates ${{\mathbf {v}}}_{j}$. For exchangeable units ${\varvec{\gamma }}$ includes an overall intercept and regression coefficients for the cluster-specific covariates in ${{\mathbf {v}}}_{j}$, and ${{\mathbf {v}}}_{j}$ includes a 1 and cluster-specific covariates.
- ${\varvec{\zeta }}_{j}$ is a vector of cluster-specific latent variables or random intercept and possibly random coefficients for the vector ${{\mathbf {z}}}_{ij}$ of item-specific and/or unit-specific covariates (that are often partly overlapping with ${\mathbf {x}}_{ij}$)

The conditional expectation of the outcome, given the covariates and latent variables, is

$$\begin{aligned} \mu _{ij} \equiv \mathrm{E}(y_{ij}|{\mathbf {x}}_{ij}, {{\mathbf {z}}}_{ij}, {{\mathbf {v}}}_j, {\varvec{\zeta }}_j) \ = \ g^{-1}(\nu _{ij}), \end{aligned}$$

and the conditional outcome distribution can be written as

2 $$\begin{aligned} p(y_{ij}|{\mathbf {x}}_{ij}, {{\mathbf {z}}}_{ij}, {{\mathbf {v}}}_j, {\varvec{\zeta }}_j)\ =\ \exp \left\{ \frac{y_{ij}\nu _{ij} -b(\nu _{ij})}{\phi } +c(y_{ij},\phi ) \right\} , \end{aligned}$$

where $\phi$ is the scale or dispersion parameter, and $b(\cdot )$ and $c(\cdot )$ are functions depending on the member of the exponential family.

We confine our treatment to three GLMs:

(i) Normal distribution (where $\phi \! = \! \sigma ^2$) and identity link for continuous outcomes, $p(y_{ij}|{\mathbf {x}}_{ij},{{\mathbf {z}}}_{ij},{{\mathbf {v}}}_{j},{\varvec{\zeta }}_j) = (\sigma \sqrt{2\pi })^{-1} \exp \{-\frac{1}{2\sigma ^2} (y_{ij}-\nu _{ij})^2 \}$ and $g(\mu _{ij})=\mu _{ij}$
(ii) Bernoulli distribution and logit link for binary outcomes,
  $$p(y_{ij}|{\mathbf {x}}_{ij},{{\mathbf {z}}}_{ij},{{\mathbf {v}}}_{j},{\varvec{\zeta }}_j) = \mu _{ij}^{y_{ij}} (1 - \mu _{ij})^{1-y_{ij}}$$ and $$g(\mu _{ij}) = \log \Bigg \{\frac{\mu _{ij}}{1-\mu _{ij}}\Bigg \}$$
(iii) Poisson distribution and log link for counts,
  $$p(y_{ij}|{\mathbf {x}}_{ij},{{\mathbf {z}}}_{ij},{{\mathbf {v}}}_{j},{\varvec{\zeta }}_j) = \exp [-\exp (\nu _{ij})]\exp (\nu _{ij})^{y_{ij}}/y_{ij}!$$ and $$g(\mu _{ij}) = \log (\mu _{ij})$$

Other members of the exponential family include the gamma and inverse-Gaussian distributions.

For simplicity we concentrate on a special case of (1) with linear predictor

3 $$\begin{aligned} \nu _{ij} \ = \ {\mathbf {x}}_{ij}^{\prime }{\varvec{\beta }}+ {{\mathbf {v}}}_{j}^{\prime }{\varvec{\gamma }}+ \zeta _j. \end{aligned}$$

It should be emphasized that this model encompasses popular latent variable or mixed models, such as generalized linear random-intercept (multilevel or hierarchical) models, and Rasch models (Rasch, 1960) and their extensions such as “explanatory” IRT (De Boeck & Wilson, 2004).

We will in the sequel also use extensions of GLMMs such as generalized linear latent and mixed models (GLLAMMs) of Rabe-Hesketh et al. (2004) and Skrondal and Rabe-Hesketh (2004).

## Cluster-Level Exogeneity and Endogeneity

Our focus is on the challenges that arise in estimation of latent variable models when there is cluster-level endogeneity. Before embarking on the challenges we must explicitly define what we mean by this term. Let ${{\mathbf {w}}}_{j}$ represent all observed covariates for cluster *j*. We say that there is *cluster-level exogeneity* if all covariates are independent of the cluster-specific intercepts; . In contrast, *cluster-level endogeneity* occurs if at least one covariate in ${{\mathbf {w}}}_{j}$ is not independent of $\zeta _j$; .

**Fig. 2:**
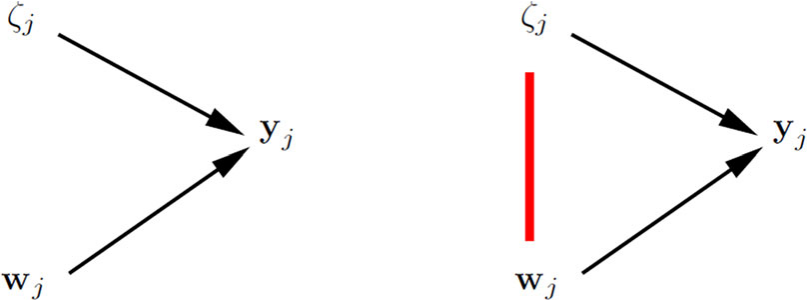
Cluster-level exogeneity (left panel) and cluster-level endogeneity (right panel).

The definitions of cluster-level exogeneity and cluster-level endogeneity are represented in the graphs in the left and right panels of Fig. 2, respectively. This kind of graph, which we find useful and will use throughout, resembles traditional directed acyclic graphs (DAGs) but nodes can represent vectors of random variables here. An arrow between two nodes means that the probability distribution of the node that the arrow points to depends on the value taken by the emanating node. The undirected arc between $\zeta _j$ and ${{\mathbf {w}}}_j$ in the right panel indicates that there is dependence between $\zeta _j$ and at least one element of ${{\mathbf {w}}}_j$.

When exploring reasons for cluster-level endogeneity in Section 12 we will rather informally rely on the *d*-separation criterion (e.g., Verma & Pearl, 1988) and the equivalent moralization criterion (Lauritzen et al., 1990) to infer cluster-level endogeneity from graphs of latent variable models, assuming “stability” or “faithfulness” to preclude dependence paths cancelling out. Sometimes we will examine likelihood contributions to show how cluster-level endogeneity arises and the consequences.

## Protective and Mitigating Estimation of Target Parameters

In this address we will focus on the performance of point estimators under cluster-level endogeneity as the number of clusters *N* becomes large, whereas the cluster sizes $n_j$ are fixed and could be small. A classical goal in statistical modeling is (weak) *consistency* of estimators for *all* model parameters as $N \, \rightarrow \, \infty$. However, this typically requires a correctly specified model, an assumption that we often deem to be naive.

A less ambitious but more realistic goal is *protective estimation* which is consistent for target parameters but possibly inconsistent for other parameters (Skrondal & Rabe-Hesketh, 2014). Our target parameters throughout will be the subset of coefficients in ${\varvec{\beta }}$ corresponding to the unit-specific covariates ${\mathbf {x}}_{ij}$ in (3). These covariates could be time-varying variables in a longitudinal study, characteristics of units *ij* in a multilevel study, or attributes of items *i* or item-subject combinations *ij* in measurement.

An even less ambitious goal is what we will refer to as *mitigating estimation* where it is likely (but not guaranteed) that estimation of the target parameters ${\varvec{\beta }}$ is less inconsistent than conventional estimation that ignores misspecification. Although mitigation in this sense cannot be formally proved, it can be made plausible by theoretical arguments and based on evidence from simulations. The hope is that “almost consistent” estimators (e.g., Laisney & Lechner, 2003) can be obtained. In reality, mitigating estimation will sometimes be the most realistic goal.

## Incidental Parameter Problems and Their Solutions

The distinction between *structural parameters* and *incidental parameters* was introduced in a seminal paper by Neyman and Scott (1948). For linear predictor (3), the structural parameters ${{\varvec{\vartheta }}}$ include ${\varvec{\beta }}$ and ${\varvec{\gamma }}$ (and $\sigma ^2$ if relevant), whereas the $\zeta _j$ are incidental parameters because their number increases in tandem with the number of clusters *N*. In econometrics a structural parameter is usually a causal parameter and for this reason Lancaster (2000) used the term “common parameter”.

Let ${{\mathbf {y}}}$ and ${{\mathbf {w}}}$ denote all outcomes and covariates for the sample, respectively. Assume that the outcomes ${{\mathbf {y}}}_j$ for the clusters are conditionally independent across clusters and the outcomes $y_{ij}$ for cluster *j* are conditionally independent, given the covariates and latent variable $\zeta _j$. The joint likelihood for the structural parameters ${{\varvec{\vartheta }}}$ and the latent variables $\zeta _1,\ldots ,\zeta _N$ (here treated as unknown parameters) becomes

4 $$\begin{aligned} p({{\mathbf {y}}}|{{\mathbf {w}}}; {{\varvec{\vartheta }}}, \zeta _1,\ldots ,\zeta _N) \ = \ \prod _{j=1}^N\prod _{i=1}^{n_j} p(y_{ij}|{{\mathbf {w}}}_j; {{\varvec{\vartheta }}}, \zeta _j). \end{aligned}$$

The incidental parameter problem (Neyman & Scott, 1948; see also Lancaster, 2000) refers to the fact that *joint maximum likelihood* (JML) estimation of both structural and incidental parameters need not be consistent for the structural parameters ${{\varvec{\vartheta }}}$ as $N \rightarrow \infty$ for fixed cluster sizes $n_j$. The problem arises because estimation of each $\zeta _j$ must often rely on a small number of units $n_j$ in the cluster. Viewing the cluster sizes as produced by $n_j = n \times m_j$, where $m_j$ has a mean of 1, the inconsistency in estimating ${{\varvec{\vartheta }}}$ for the models considered here is of order $n^{-1}$ (e.g., Arellano & Hahn, 2007). Note that JML estimation has also been referred to as unconditional maximum likelihood estimation (e.g., Wright & Douglas, 1977) and unconstrained maximum likelihood estimation (e.g., de Leeuw & Verhelst, 1986) in psychometrics.

There is no incidental parameter problem when the joint likelihood can be factorized into two components, one just containing structural parameters and the other just incidental parameters. Such likelihood orthogonality (e.g., Lancaster, 2000) occurs for linear predictor (3) with (a) identity link and normal conditional distribution (e.g., Chamberlain, 1980) and (b) log link and Poisson conditional distribution (e.g., Cameron & Trivedi, 1999). For these models JML estimation is consistent for ${\varvec{\beta }}$ when $N \rightarrow \infty$ for fixed $n_{j}$.

In general, consistent JML estimation can be achieved under a double-asymptotic scheme where both the number of units per cluster increases $n \rightarrow \infty$ and the number of clusters increases $N \rightarrow \infty$. In psychometrics, a classical result is that ${\widehat{{\varvec{\beta }}}}^{\scriptscriptstyle \mathrm JML}$ is consistent for the Rasch model in this case if $\frac{N}{n} \rightarrow \infty$ (Haberman, 1977). Based on simulation evidence, Greene (2004) observed that ${\widehat{{\varvec{\beta }}}}^{\scriptscriptstyle \mathrm JML}$ appears to be consistent for many latent variable models used in econometrics under double asymptotics. However, appealing to double asymptotics is unconvincing when *n* is not large.

For the simple Rasch model, the inconsistency of JML estimation can be derived and corrected. When $n=2$, Andersen (1973a) showed that $p\text{ lim } \ {\widehat{{\varvec{\beta }}}}^{\scriptscriptstyle \mathrm JML} = 2{\varvec{\beta }}$ as $N \rightarrow \infty$, so $\frac{1}{2} \, {\widehat{{\varvec{\beta }}}}^{\scriptscriptstyle \mathrm JML}$ is consistent. For general *n*, Wright and Douglas (1977) observed that the finite sample bias is approximately $\frac{1}{n-1} \, {{\varvec{\beta }}}^{}$ and discussed the bias correction $\frac{n-1}{n} \, {\widehat{{\varvec{\beta }}}}^{\scriptscriptstyle \mathrm JML}$. Andersen (1980, Theorem 6.1) stated the same result for inconsistency. For more complex models, methods that reduce inconsistency from order $n^{-1}$ to $n^{-2}$ are discussed in Arellano and Hahn (2007). For instance, a *modified profile likelihood* where the incidental parameters $\zeta _{j}$ are “profiled out” of the joint likelihood has been used for models with linear predictors such as (3) by Bellio and Sartori (2006) and Bartolucci et al. (2016). This approach can produce mitigating estimation.

An approach usually called *marginal maximum likelihood* (MML) estimation in psychometrics is the most popular for linear predictor (3). Here, $\zeta _{j}$ is treated as a random variable and “integrated out” of the joint likelihood, as proposed in early work by Kiefer and Wolfowitz (1956). Note that the statistical literature typically refers to this likelihood as *integrated* and that their marginal likelihood “transforms away” incidental parameters (e.g., Kalbfleisch & Sprott, 1970). The terms unconditional maximum likelihood estimation (e.g., Bock & Lieberman, 1970) and, simply, maximum likelihood estimation (e.g., Holland, 1990) have also been used in psychometrics. Under assumptions including cluster-level exogeneity, MML is consistent for all model parameters.

Alternatively, *conditional maximum likelihood* (CML) estimation can be used where $\zeta _{j}$ is treated as a fixed parameter and “conditioned out” of the joint likelihood. The idea of CML estimation was discussed already by Bartlett (1936, 1937a), the eminent British statistician whose name is associated with factor scores in psychometrics (e.g., Bartlett, 1937b). We will see that CML can yield protective estimation of ${\varvec{\beta }}$ under cluster-level endogeneity.

## Marginal Maximum Likelihood (MML) Estimation

In marginal maximum likelihood (MML) estimation the cluster-specific intercept $\zeta _{j}$ is treated as a *random variable* in estimation. The following assumptions are usually made:

- [A.1] Cluster independence: $$p({{\mathbf {y}}}|{{\mathbf {w}}},\zeta _1,\ldots ,\zeta _N; {{\varvec{\vartheta }}}) \ = \ \prod _{j=1}^{N} p({{\mathbf {y}}}_{j}|{{\mathbf {w}}}_{j},\zeta _j; {{\varvec{\vartheta }}})$$
- [A.2] Conditional unit independence: $p({{\mathbf {y}}}_{j}|{{\mathbf {w}}}_j,\zeta _j; {{\varvec{\vartheta }}}) \ = \ \prod _{i=1}^{n_j} p(y_{ij}|{{\mathbf {w}}}_{j},\zeta _j; {{\varvec{\vartheta }}})$
- [A.3] Strict exogeneity conditional on the latent variable: $p(y_{ij}|{{\mathbf {w}}}_{j},\zeta _{j}; {{\varvec{\vartheta }}}) = \ p(y_{ij}|{\mathbf {x}}_{ij},{{\mathbf {v}}}_{j},\zeta _{j}; {{\varvec{\vartheta }}})$; i.e., given the latent variable, the outcome for a unit only depends on covariates for that unit
- [A.4] Correct conditional distribution: $p(y_{ij}|{\mathbf {x}}_{ij},{{\mathbf {v}}}_{j},\zeta _{j}; {{\varvec{\vartheta }}})$ follows (2) and (3)
- [A.5] Cluster-level exogeneity: $p(\zeta _{j}|{{\mathbf {w}}}_{j}) \ = \ p(\zeta _{j})$
- [A.6] Latent variable normality: $p(\zeta _j) \ = \ \phi (\zeta _j;0,\psi )$; a normal density with zero expectation and variance $\psi$

Using [A.2]-[A.6], the marginal likelihood contribution of cluster *j* simplifies in the following way:

$$\begin{aligned} {{{\mathcal {L}}}}_j^{\scriptscriptstyle \mathrm{MML}} \equiv p({{\mathbf {y}}}_{j}|{{\mathbf {w}}}_{j}; {{\varvec{\vartheta }}})= & {} \int _{\zeta _{j}} p({{\mathbf {y}}}_{j}|{{\mathbf {w}}}_{j},\zeta _{j};{{\varvec{\vartheta }}}) \, p(\zeta _{j}|{{\mathbf {w}}}_{j}) \, \mathrm{d} \zeta _{j} \\= & {} \int _{\zeta _{j}} \left\{ \prod _{i=1}^{n_j} p(y_{ij}|{\mathbf {x}}_{ij},{{\mathbf {v}}}_{j},\zeta _{j}; {{\varvec{\vartheta }}}) \right\} \phi (\zeta _j;0,\psi ) \, \mathrm{d} \zeta _{j}, \end{aligned}$$

where we see that $\zeta _j$ is marginalized over or integrated out of the joint likelihood.

$\phi (\zeta _j;0,\psi )$ can be interpreted as the density of a cluster-specific disturbance in a data-generating mechanism or as a superpopulation density of clusters in survey sampling. That the $\zeta _j$ are independently and identically distributed random variables can be motivated by exchangeability of the clusters (e.g., Draper, 1995).

Using [A.1], MML estimation proceeds by maximizing the likelihood ${{{\mathcal {L}}}}^{\scriptscriptstyle \mathrm{MML}} = \prod _{j=1}^{N} {{{\mathcal {L}}}}_j^{\scriptscriptstyle \mathrm{MML}}$ w.r.t. ${\varvec{\beta }}$, ${\varvec{\gamma }}$ and $\psi$ (and $\sigma ^2$ if relevant). If the above assumptions are satisfied, MML estimators are consistent as $N \rightarrow \infty$ for fixed $n_{j}$ for *all* parameters under appropriate regularity conditions (e.g., Butler & Louis, 1997). Importantly, standard MML estimation becomes inconsistent, possibly severely, for all link functions when the exogeneity assumptions are violated. As we will see momentarily, inconsistency due to violation of [A.5] can arise because MML estimation of ${\varvec{\beta }}$ exploits both within-cluster and between-cluster information, and the latter can be contaminated by cluster-level endogeneity.

### MML Estimation for Identity Link and Normal Distribution

It is instructive to consider the linear predictor (3) with an identity link and a normal conditional distribution that can be written as

5 $$\begin{aligned} y_{ij} \ = \ {\mathbf {x}}_{ij}^{\prime }{\varvec{\beta }}+ {{\mathbf {v}}}_{j}^{\prime }{\varvec{\gamma }}+ \zeta _{j} + \epsilon _{ij}, \end{aligned}$$

where $\epsilon _{ij}$ is an additive normally distributed unit-level error term, $p(\epsilon _{ij}|{\mathbf {x}}_{ij},{{\mathbf {v}}}_{j},\zeta _{j}) = \phi (\epsilon _{ij}; 0,\sigma ^2)$.

For identity links the assumptions given above are stricter than necessary for consistent MML estimation. First, for identity and log links, [A.4] can be replaced by the more lenient assumption that $\mu _{ij}$ is correctly specified (and the domain of $y_{ij}$ for the assumed exponential family distribution encompasses the domain of the correct distribution). This extends the idea of pseudo maximum likelihood (PML) estimation (Gourieroux et al., 1984) to what we may call pseudo marginal maximum likelihood estimation in the latent variable setting. For the identity link, [A.3], [A.4], and [A.5] can be replaced by (5) with a weaker set of assumptions where normality is relaxed for $\zeta _{j}$ and $\epsilon _{ij}$, $\mathrm{E}(\epsilon _{ij}| {{\mathbf {w}}}_{j}, \zeta _{j}) = 0$ (a mean-independence version of “unit-level exogeneity”), and $\mathrm{E}(\zeta _{j}| {{\mathbf {w}}}_{j}) = 0$ (e.g., Wooldridge, 2010, p. 292). Second, for the identity link, consistent estimation of ${\varvec{\beta }}$ and ${\varvec{\gamma }}$ neither requires assumption [A.2], see Zeger et al. (1988), nor assumption [A.6], see Verbeke and Lesaffre (1997).

We now outline how MML estimation relies on both between-cluster and within-cluster information, for simplicity omitting ${{\mathbf {v}}}_{j}^{\prime }{\varvec{\gamma }}$ from model (5) and letting $n_{j}=n$. The total sum of squares of $y_{ij}$ can then be decomposed into two contributions:

6 $$\begin{aligned} T_{yy} = \mathop {\Sigma }\limits _{j=1}^N \mathop {\Sigma }\limits _{i=1}^{n} (y_{ij}-{\overline{y}}_{\cdot \cdot })^2 \ = \ {\mathop {\Sigma }\limits _{j=1}^N \mathop {\Sigma }\limits _{i=1}^{n} (y_{ij}-{\overline{y}}_{\cdot j})^2} + {\mathop {\Sigma }\limits _{j=1}^N \mathop {\Sigma }\limits _{i=1}^{n} ({\overline{y}}_{\cdot j}-{\overline{y}}_{\cdot \cdot })^2} = W_{yy} + B_{yy}, \end{aligned}$$

where $W_{yy}$ represents the within-cluster variation and $B_{yy}$ the between-cluster variation. We use similar decompositions of $T_{{\mathbf {x}}{\mathbf {x}}}$ into $W_{{\mathbf {x}}{\mathbf {x}}}$ and $B_{{\mathbf {x}}{\mathbf {x}}}$, and $T_{{{\mathbf {x}}} y}$ into $W_{{{\mathbf {x}}} y}$ and $B_{{{\mathbf {x}}} y}$. For known variance components $\psi$ and $\sigma ^2$, the MML estimator is the generalized least squares (GLS) estimator that Maddala (1971) shows can be expressed as

7 $$\begin{aligned} {\widehat{{\varvec{\beta }}}}^{\scriptscriptstyle \mathrm{GLS}} \ = \ (W_{{\mathbf {x}}{\mathbf {x}}} + \omega B_{{\mathbf {x}}{\mathbf {x}}})^{-1} (W_{{{\mathbf {x}}} y} + \omega B_{{{\mathbf {x}}} y}), \end{aligned}$$

where $\omega \equiv \frac{\sigma ^2}{\sigma ^2+ n \psi }$ is the weight given to the between-cluster variation. The GLS estimator in essence combines the between-cluster and within-cluster estimators of ${\varvec{\beta }}$ by weighting them in inverse proportion to their respective variances. Fuller and Battese (1973) demonstrate that the GLS estimator can be obtained by using ordinary least squares (OLS) for the transformed data ${\widetilde{y}}_{ij} = y_{ij} - \theta {\overline{y}}_{\cdot j}$ and ${\widetilde{{\mathbf {x}}}}_{ij} = {\mathbf {x}}_{ij} - \theta {\overline{{\mathbf {x}}}}_{\cdot j}$, where $\theta = 1-\sqrt{\omega }$. The probability limit of ${\widehat{{\varvec{\beta }}}}^{\scriptscriptstyle \mathrm{GLS}}$ as $N \rightarrow \infty$ can be expressed as

8 $$\begin{aligned} p\text{ lim } \ {\widehat{{\varvec{\beta }}}}^{\scriptscriptstyle \mathrm{GLS}} \ = \ {\varvec{\beta }}+ \omega \, \Sigma _{{\widetilde{{\mathbf {x}}}}}^{-1}\Sigma _{{{\mathbf {x}}} \zeta }, \end{aligned}$$

where $\Sigma _{{\widetilde{{\mathbf {x}}}}}$ is the covariance matrix of ${\widetilde{{\mathbf {x}}}}_{ij}$, and $\Sigma _{{{\mathbf {x}}} \zeta }$ is the covariance matrix of ${\mathbf {x}}_{ij}$ with $\zeta _{j}$. Importantly, the estimator is inconsistent if cluster-level exogeneity [A.5] is violated because this implies that $\Sigma _{{{\mathbf {x}}} \zeta } \ne {{\mathbf {0}}}$.

Analytical integration is trivial for models with conjugate latent variable densities for which ${{{\mathcal {L}}}}^{\scriptscriptstyle \mathrm{MML}}$ can be written in closed form. For a GLMM with linear predictor (3) and identity link, ${{{\mathcal {L}}}}^{\scriptscriptstyle \mathrm{MML}}$ simply takes the form of the (ordinary) likelihood of a multivariate normal regression model with $\mathrm{E}({{\mathbf {y}}}_j \! \mid \! {{\mathbf {w}}}_j) = {\mathbf {X}}_{j}{\varvec{\beta }}+ ({{\mathbf {1}}}_{n_{j}} \! \otimes {{\mathbf {v}}}_{j}^{\prime }){\varvec{\gamma }}$ (where ${\mathbf {x}}_{1j}^{\prime },\ldots ,{\mathbf {x}}_{n_{j}j}^{\prime }$ are the rows of ${\mathbf {X}}_{j}$) and $\mathrm{Var}({{\mathbf {y}}}_j \! \mid \! {{\mathbf {w}}}_j) = \psi {{\mathbf {1}}}_{n_{j}}{{\mathbf {1}}}_{n_{j}}^{\prime } + \sigma {{\mathbf {I}}}_{n_{j}}$. MML estimation of linear mixed models when variance components are unknown is discussed by Laird and Ware (1982) using the EM algorithm and by Goldstein (1986) using iterative generalized least squares (IGLS).

### MML Estimation for Logit Link and Bernoulli Distribution or Log Link and Poisson Distribution

Because $\mu _{ij}$ is nonlinear in $\zeta _{j}$ for log and logit links, ${{{\mathcal {L}}}}^{\scriptscriptstyle \mathrm{MML}}$ cannot be expressed in closed form and maximization is usually based on numerical integration (e.g., Rabe-Hesketh et al., 2005) or Monte Carlo integration (e.g., Booth & Hobert, 1999).

Unfortunately, it is difficult to assess the normality assumption for the latent variables [A.6] for logit links. However, MML estimators for regression coefficients are almost consistent if [A.6] is violated, whereas estimators for intercepts and random effect variances can be severely inconsistent if the correct latent variable density is highly skewed (e.g., Neuhaus et al., 1992). The threats posed by violation of the cluster-level exogeneity assumption [A.5] persist.

## Conditional Maximum Likelihood (CML) Estimation

We retain assumptions [A.1]-[A.4] stated for MML estimation but now relax assumptions [A.5] and [A.6] regarding the latent variable distribution.

Using the exponential family distribution (2) in conjunction with linear predictor (3), we obtain

$$\begin{aligned} p({{\mathbf {y}}}_{j}|{{\mathbf {w}}}_j; {{\varvec{\vartheta }}}, \zeta _j) \ \propto \ \exp \left\{ {\varvec{\beta }}^{\prime }\sum _{i=1}^{n_j} {\mathbf {x}}_{ij}y_{ij} + (\zeta _j \! + \! {\varvec{\gamma }}^{\prime }{{\mathbf {v}}}_{j})\sum _{i=1}^{n_j} y_{ij} - \sum _{i=1}^{n_j} b(\nu _{ij}) \right\} . \end{aligned}$$

It follows from the Neyman-Fisher factorization theorem (e.g., Pawitan, 2001, Theorem 3.1) that for known ${\varvec{\beta }}$, the cluster-specific sumscore of the outcomes, $\sum _{i=1}^{n_j} y_{ij}$, is a sufficient statistic for $\zeta _j + {\varvec{\gamma }}^{\prime }{{\mathbf {v}}}_{j}$ (and that $\sum _{i=1}^{n_j} {\mathbf {x}}_{ij}y_{ij}$ is a sufficient statistic for ${\varvec{\beta }}$).

The conditional likelihood contribution of cluster *j*, given $\tau _j \equiv \sum _{i=1}^{n_j} y_{ij}$, can be expressed as

$$\begin{aligned} {{{\mathcal {L}}}}_j^{\scriptscriptstyle \mathrm{CML}} \equiv p({{\mathbf {y}}}_j | \tau _j, {{\mathbf {w}}}_{j}; {{\varvec{\vartheta }}}, \zeta _j) \ = \ \frac{\prod _{i=1}^{n_j} p(y_{ij}|{\mathbf {x}}_{ij},{{\mathbf {v}}}_{j}; {{\varvec{\vartheta }}}, \zeta _j) }{ p(\sum _{i=1}^{n_j} y_{ij} | {\mathbf {x}}_{ij},{{\mathbf {v}}}_{j}; {{\varvec{\vartheta }}}, \zeta _j)}. \end{aligned}$$

Importantly, the cluster-specific term $\zeta _j + {{\mathbf {v}}}_{j}^{\prime }{\varvec{\gamma }}$ cancels out of the numerator and denominator of ${{{\mathcal {L}}}}_j^{\scriptscriptstyle \mathrm{CML}}$ and the latent variable assumptions [A.5] and [A.6] are therefore no longer required.

CML estimation proceeds by maximizing the conditional likelihood ${{{\mathcal {L}}}}^{\scriptscriptstyle \mathrm{CML}} = \prod _{j=1}^{N} {{{\mathcal {L}}}}_j^{\scriptscriptstyle \mathrm{CML}}$ w.r.t. ${{\varvec{\vartheta }}}$, where ${{\varvec{\vartheta }}}$ is a vector containing ${\varvec{\beta }}$ (and $\sigma ^2$ if relevant) here. If the above assumptions are satisfied together with appropriate regularity conditions, CML estimators are consistent as $N \rightarrow \infty$ for fixed $n_j$ (e.g., Andersen, 1970, 1973a).

The conditional likelihood ${{{\mathcal {L}}}}^{\scriptscriptstyle \mathrm{CML}}$ is almost invariably derived by treating the cluster-specific latent variable $\zeta _j$ as a fixed parameter, although Sartori and Severini (2004) show that the same ${{{\mathcal {L}}}}^{\scriptscriptstyle \mathrm{CML}}$ results if $\zeta _j$ is treated as a random variable. $\zeta _j$ is usually interpreted as fixed when using CML estimation in psychometrics (e.g., Holland, 1990), whereas the “fixed effects framework” of econometrics interprets $\zeta _j$ as a random variable that can have arbitrary dependence with the covariates (e.g., Wooldridge, 2010, p. 286).

In contrast to MML estimation, CML estimation is based on solely within-cluster information. A great advantage of CML estimation is therefore that it is protective for the target parameters ${\varvec{\beta }}$ if [A.1]-[A.4] are satisfied, regardless of the latent variable distribution and even if there is cluster-level endogeneity. It is usually not recognized that CML estimation also has a role to play under exogeneity because performance does not rely on ${{\mathbf {v}}}_{j}^{\prime }{\varvec{\gamma }}$ being the correct specification of the functional form for ${{\mathbf {v}}}_j$.

A cost of CML estimation is that it can be inefficient because it just exploits within-cluster information. Inefficiency is particularly acute when there is little within-cluster variation in the unit-specific covariates. Hence, CML estimation may have larger mean squared errors for estimating ${\varvec{\beta }}$ than MML estimation, even under cluster-level endogeneity (e.g., Palta & Yao, 1991). Also, using CML estimation to remove cluster-specific slopes can lead to pronounced inefficiency.

CML estimation is primarily useful if the coefficients ${\varvec{\beta }}$ of unit-specific covariates are the target parameters because the coefficients ${\varvec{\gamma }}$ of cluster-specific covariates and the covariance parameters of random effects cannot be estimated. In our view, this may actually be beneficial because these parameters are inconsistently estimated by standard MML if there is cluster-level endogeneity.

Interactions between cluster-specific and unit-specific covariates become elements of ${\varvec{\beta }}$ and can be estimated by CML for models with cluster-specific intercepts. For instance, the treatment-by-time interaction is often the target parameter in longitudinal data with time-invariant treatments. In models with cluster-specific random coefficients, the vectors ${\mathbf {x}}_{ij}$ and ${{\mathbf {z}}}_{ij}$ often include common variables and the corresponding elements of ${\varvec{\beta }}$ are conditioned away by CML. Hence, the treatment-by-time interaction parameter cannot be estimated by CML in a model with cluster-specific slopes of time (e.g., Liang & Zeger, 2000).

In some situations covariate measurement error or misclassification problems can be more serious for CML than MML estimation (e.g., Griliches & Hausman, 1986; Frisell et al., 2012). However, CML estimation is immune to such problems for ${{\mathbf {v}}}_j$ and we will later show that cluster-specific covariate measurement error for ${\mathbf {x}}_{ij}$ is handled.

We now show the form taken by the conditional likelihood contribution ${{{\mathcal {L}}}}_j^{\scriptscriptstyle \mathrm{CML}}$ for identity, logit and log links.

### CML Estimation for Identity Link and Normal Distribution

Using that the sum of conditionally independent normally distributed random variables has a normal distribution, the conditional likelihood contribution for cluster *j* in model (5) becomes (e.g., Chamberlain, 1980)

$$\begin{aligned} {{{\mathcal {L}}}}_j^{\scriptscriptstyle \mathrm{CML}} \ = \ n_j^{1/2} (\sqrt{2\pi }\sigma )^{-(n_j - 1)} \exp \left\{ -\frac{1}{2\sigma ^2}\sum _{i=1}^{n_j} \left[ (y_{ij} \! - \! {\overline{y}}_{\cdot j}) \! - \! ({\mathbf {x}}_{ij} \! - \! {\overline{{\mathbf {x}}}}_{\cdot j})^{\prime }{\varvec{\beta }}\right] ^2\right\} . \end{aligned}$$

We see that the cluster-level component $\zeta _j \! + \! {{\mathbf {v}}}_{j}^{\prime }{\varvec{\gamma }}$ has cancelled out of ${{{\mathcal {L}}}}_j^{\scriptscriptstyle \mathrm{CML}}$ and that a cluster does not contribute to estimation of ${\varvec{\beta }}$ if the ${\mathbf {x}}_{ij}$ do not vary within the cluster.

For the identity link, assumption [A.2] is not required for consistent estimation, and [A.4] can be replaced by $\mathrm{Cor}[({\mathbf {x}}_{ij} \! - \! {\overline{{\mathbf {x}}}}_{\cdot j}),(\epsilon _{ij} \! - \! {\overline{\epsilon }}_{\cdot j})]={{\mathbf {0}}}$. The effects of violating this zero-correlation assumption can in some cases be more severe for CML than MML estimation (e.g., Bound & Solon, 1999; Frisell et al., 2012). This can be addressed by using instrumental variables (IV) estimation if plausible instruments are available (e.g., Ebbes et al., 2004). Chamberlain (1984, 1985) proposes tests of [A.3] for the identity link (and the probit link) and Sjölander et al. (2016) derive the inconsistency produced by various violations of [A.2] and [A.3] for the identity link (and the logit link for some instances) in sibling designs.

For the identity link there are several alternative ways of implementing CML estimation of ${\varvec{\beta }}$. We will briefly describe them below because they provide insight into basic features of CML estimation and its connection to other estimation methods in this particular case.

#### Cluster-Mean Centering and Maximum “Marginal” Likelihood Estimation in Statistics

Consider the cluster-mean centered or within-cluster model

9 $$\begin{aligned} y_{ij} - {\overline{y}}_{\cdot j} \ = \ ({\mathbf {x}}_{ij}-{\overline{{\mathbf {x}}}}_{\cdot j})^{\prime }{\varvec{\beta }}+ (\epsilon _{ij} - {\overline{\epsilon }}_{\cdot j}), \end{aligned}$$

which is an example of a “working model” derived from the assumed data-generating mechanism. We see that $\zeta _j$ is swept out of the model and any misspecification related to $\zeta _j$ is therefore immaterial. However, ${{\mathbf {v}}}_{j}^{\prime }{\varvec{\gamma }}$ (and actually any cluster-specific function of ${{\mathbf {v}}}_j$) is also swept out which precludes estimation of ${\varvec{\gamma }}$. On the other hand, MML estimation of ${\varvec{\gamma }}$ is inconsistent if either ${\mathbf {x}}_{ij}$ or ${{\mathbf {v}}}_j$ are cluster-level endogenous as is estimation of both ${\varvec{\beta }}$ and ${\varvec{\gamma }}$ if the functional form ${{\mathbf {v}}}_{j}^{\prime }{\varvec{\gamma }}$ is incorrect.

The maximum likelihood (ML) estimator of ${\varvec{\beta }}$ in this model coincides with the CML estimator, which it not surprising given the resemblance of (9) with the argument of the exponential function in ${{{\mathcal {L}}}}_j^{\scriptscriptstyle \mathrm{CML}}$. Furthermore, the CML estimator of ${\varvec{\beta }}$ is the *within-cluster* ordinary least squares (OLS) estimator

$$\begin{aligned} {\widehat{{\varvec{\beta }}}}^{\scriptscriptstyle \mathrm{CML}} \ = \ W_{{\mathbf {x}}{\mathbf {x}}}^{-1}W_{{{\mathbf {x}}} y}, \end{aligned}$$

which is the special case of (7) where $\omega =0$ and between-cluster information is hence ignored.

Using standard terminology in statistics (e.g., Pawitan, 2001), the likelihood based on (9) can be viewed as a *marginal* likelihood that “transforms away” the nuisance parameters (here the incidental parameters $\zeta _j$) by considering the implied model for the deviations from cluster means $y_{ij} - {\overline{y}}_{\cdot j}$. The canonical example of a marginal likelihood in the statistical sense is the restricted or residual likelihood of Patterson and Thompson (1971) for variance components in linear mixed models, where regression coefficients are nuisance parameters. Recall that this meaning of “marginal” is different from that in psychometrics where it refers to a likelihood where random $\zeta _j$ are “integrated out”.

Goetgeluk and Vansteelandt (2008) use cluster-mean centering to consistently estimate ${\varvec{\beta }}$ under cluster-level endogeneity by conditional generalized estimating equations (CGEE), an estimator that can also be used for log links.

#### Cluster-Specific Dummy Variables and JML Estimation

Alternatively, we can use JML estimation for ${\varvec{\beta }}$ in a working model that includes dummy or indicator variables with fixed cluster-specific coefficients $\zeta _j$

10 $$\begin{aligned} y_{ij} \ = \ {\mathbf {x}}_{ij}^{\prime }{\varvec{\beta }}+ \sum \nolimits {_{r=1}^N} \, d_{rj} \zeta _r + \epsilon _{ij}, \end{aligned}$$

where the $d_{rj}$ take the value 1 if $r\!=\!j$ and 0 otherwise. Note that ${{\mathbf {v}}}_{j}^{\prime }{\varvec{\gamma }}$ is omitted because ${{\mathbf {v}}}_j$ is collinear with the dummy variables $d_{rj}$. JML estimation of ${\varvec{\beta }}$ can simply proceed by using OLS to estimate (10).

The use of a fixed parameter for each cluster explains why CML and related estimators are often referred to as *fixed-effects* estimators in econometrics. In that literature, MML and related estimators that assume cluster-level exogeneity are referred to as *random-effects* estimators, although the estimands are not the random effects.

By explicitly controlling for clusters in this way we are estimating pure within-cluster effects. The clusters are said to act as their own controls, and estimation is therefore immune to cluster-level endogeneity. As mentioned earlier, there is no incidental parameter problem in this case and ${\widehat{{\varvec{\beta }}}}^{\scriptscriptstyle \mathrm JML} = {\widehat{{\varvec{\beta }}}}^{\scriptscriptstyle \mathrm CML}$ is consistent as $N \rightarrow \infty$ for fixed $n_j$. Consistency of ${\widehat{\zeta }}_j^{\scriptscriptstyle \mathrm JML}$ requires a double-asymptotic scheme where both $n_j \rightarrow \infty$ and $N \rightarrow \infty$.

#### Auxiliary Linear Projection and MML Estimation

In the Mundlak-device (Mundlak, 1978) the cluster means ${\overline{{\mathbf {x}}}}_{\cdot j}$ of the unit-specific covariates ${\mathbf {x}}_{ij}$ are included in the model. This can be viewed as handling violation of [A.5] by considering an auxiliary linear projection

11 $$\begin{aligned} \zeta _j \ = \ {\overline{{\mathbf {x}}}}_{\cdot j}^{\prime }{{\varvec{\delta }}}+ u_j, \end{aligned}$$

where $\mathrm{Cov}({\overline{{\mathbf {x}}}}_{\cdot j},u_j)={{\mathbf {0}}}$ per construction. Substituting the linear projection in (5), we obtain

$$\begin{aligned} y_{ij} \ = \ {\mathbf {x}}_{ij}^{\prime }{\varvec{\beta }}+ {\overline{{\mathbf {x}}}}_{\cdot j}^{\prime }{{\varvec{\delta }}}+ {{\mathbf {v}}}_{j}^{\prime }{\varvec{\gamma }}+ u_j + \epsilon _{ij}, \end{aligned}$$

which can be expressed as the following model that is estimated in the “hybrid method” of Allison (2009):

12 $$\begin{aligned} y_{ij} \ = \ ({\mathbf {x}}_{ij}^{\prime } \! - \! {\overline{{\mathbf {x}}}}_{\cdot j}^{\prime }){\varvec{\beta }}+ {\overline{{\mathbf {x}}}}_{\cdot j}^{\prime }({\varvec{\beta }}\! + \! {{\varvec{\delta }}}) + {{\mathbf {v}}}_{j}^{\prime }{\varvec{\gamma }}+ u_j + \epsilon _{ij}. \end{aligned}$$

For the identity link, MML estimation (or ML/OLS estimation treating the composite error terms $u_j + \epsilon _{ij}$ as independent) of these working models produces the consistent CML estimator of ${\varvec{\beta }}$, even if the linear projection does not coincide with the correct auxiliary statistical model or “data-generating mechanism”. Contrary to common belief (e.g., Allison, 2009), the hybrid method is inconsistent for ${\varvec{\gamma }}$ (and $\psi$) even if ${{\mathbf {v}}}_{j}$ is cluster-level exogenous because ${{\varvec{\delta }}}$ absorbs some of the effects of the cluster-level covariates ${{\mathbf {v}}}_{j}$ (Castellano et al., 2014).

#### Including Deviations from Cluster Means and E-Estimation

In the vector version of (12) for cluster *j*, the matrix of cluster mean deviations ${\mathbf {X}}_j - {{\mathbf {1}}}_{n_j} \! \otimes {\overline{{\mathbf {x}}}}_{\cdot j}^{\prime }$ is orthogonal to ${{\mathbf {1}}}_{n_j} \! \otimes {{\mathbf {v}}}_{j}^{\prime }$ and ${{\mathbf {1}}}_{n_j}\zeta _j$, so the CML estimator for ${\varvec{\beta }}$ is obtained by estimating the simplified working model

$$\begin{aligned} y_{ij} \ = \ ({\mathbf {x}}_{ij}^{\prime } \! - \! {\overline{{\mathbf {x}}}}_{\cdot j}^{\prime }){\varvec{\beta }}+ \epsilon _{ij}, \end{aligned}$$

by ML/OLS.

This can be viewed as a variant of *E*-estimation of ${\varvec{\beta }}$ (e.g., Robins et al., 1992). Here, specification of a correct model for the association between the outcome and possibly unknown cluster-level covariates in the “outcome model” is avoided by breaking the correlation between the unit-level covariates (“exposures” ${\mathbf {x}}_{ij}$) and cluster-level variables $\zeta _j$ and ${{\mathbf {v}}}_j$ in the “exposure model” through the inclusion of ${\overline{{\mathbf {x}}}}_{\cdot j}$ (see also Goetgeluk & Vansteelandt, 2008).

#### Using Deviations from Cluster Means as Instrumental Variables

Because ${\mathbf {x}}_{ij}-{\overline{{\mathbf {x}}}}_{\cdot j}$ is correlated with ${\mathbf {x}}_{ij}$ whereas ${\mathbf {X}}_j - {{\mathbf {1}}}_{n_j} \! \otimes {\overline{{\mathbf {x}}}}_{\cdot j}^{\prime }$ is orthogonal to ${{\mathbf {1}}}_{n_j}\zeta _j$ by construction, ${\mathbf {x}}_{ij}-{\overline{{\mathbf {x}}}}_{\cdot j}$ can serve as instrumental variable for ${\mathbf {x}}_{ij}$ in the outcome model (5). Instrumental variables (IV) estimators, such as two-stage least squares (2SLS), are then identical to the CML estimator for ${\varvec{\beta }}$.

### CML Estimation for Logit Link and Bernoulli Distribution

We now consider linear predictor (3) with a logit link and a Bernoulli conditional distribution. The conditional likelihood contribution for cluster *j* can be expressed as (e.g., Chamberlain, 1980)

13 $$\begin{aligned} {{{\mathcal {L}}}}_j^{\scriptscriptstyle \mathrm{CML}} \ = \ \frac{ \prod _{i=1}^{n_j} \exp ( {\mathbf {x}}_{ij}^{\prime }{\varvec{\beta }})^{y_{ij}} }{ \sum _{{{\mathbf {d}}}\in {{{\mathcal {B}}}}_j} \prod _{i=1}^{n_j} \exp ( {\mathbf {x}}_{ij}^{\prime }{\varvec{\beta }})^{d_{i}}}. \end{aligned}$$

Here,

14 $$\begin{aligned} {{{\mathcal {B}}}}_j = \left\{ {{\mathbf {d}}}\! = \! (d_1,\ldots ,d_{n_j})^{\prime }\, : d_{i} \! \in \! \{ 0,1 \}, \ \sum \nolimits {_{i=1}^{n_j}} \, d_{i}\!=\! \tau _{j} \right\} \end{aligned}$$

is the set of all $n_j \atopwithdelims ()\tau _j$ permutations of zeros and ones whose sum equals $\tau _{j}$, the observed value of the sufficient statistic for $\zeta _j$.

We note that $\zeta _j \! + \! {{\mathbf {v}}}_{j}^{\prime }{\varvec{\gamma }}$ has cancelled out of ${{{\mathcal {L}}}}_j^{\scriptscriptstyle \mathrm{CML}}$. Also, a cluster does not contribute to the conditional likelihood if its outcomes $y_{ij}$ are all 0 or all 1, or ${\mathbf {x}}_{ij}$ does not vary in the cluster. CML estimation appears computationally demanding but is feasible even for large $n_j$ by using recursive algorithms (e.g., Howard, 1972; Gustafsson, 1980) or Markov chain Monte Carlo (e.g., Rice, 2004). For very large $n_j$, approximations can be based on composite conditional likelihoods (e.g., Liang, 1987) or random sampling of permutations in ${{{\mathcal {B}}}}_j$ (e.g., D’Haultfæuille & Iaria, 2016). For $n \! = \! 2$, ${{{\mathcal {L}}}}_j^{\scriptscriptstyle \mathrm{CML}}$ simplifies to the standard likelihood contribution of a logistic regression model with binary outcome equal to 1 if $(y_{1j} \! = \! 0, y_{2j} \! = \! 1)$ and equal to 0 if $(y_{1j} \! = \! 1, y_{2j} \! = \! 0)$, and with covariates ${\mathbf {x}}_{2j} \! - \! {\mathbf {x}}_{1j}$.

Several other models have likelihood contributions that take a similar form as (13):

(i) Case-control studies: ${{{\mathcal {L}}}}_j^{\scriptscriptstyle \mathrm{CML}}$ corresponds to the conditional likelihood contribution for matched set *j* in conditional logistic regression for matched retrospective case-control designs, were the indicator $y_{ij}$ takes the value 1 if unit *i* is one of a fixed number of $\tau _{j}$ cases and 0 if unit *i* is one of $n_j-\tau _{j}$ controls. ${{{\mathcal {L}}}}_j^{\scriptscriptstyle \mathrm{CML}}$ then represents the conditional probability of the $n_j$ observed covariate vectors in set *j*, given all potential allocations of the covariate vectors to cases and controls (e.g., Prentice & Breslow, 1978).
(ii) Survival analysis with ties: ${{{\mathcal {L}}}}_j^{\scriptscriptstyle \mathrm{CML}}$ corresponds to the “discrete” or “exact” partial likelihood contribution for the *j*th ordered survival time in a Cox proportional-hazards model with tied survival times (Cox, 1972). At the *j*th survival time, the indicator $y_{ij}$ takes the value 1 if unit *i* is one of the $\tau _{j}$ units in the risk set who experienced the event and ${{{\mathcal {B}}}}_j$ is defined as in (14) for the $n_j$ units in the risk set. When there are no ties, the standard partial likelihood contribution for an event occurring at the *j*th survival time can be expressed as
  15 $$\begin{aligned} \frac{ \exp ( {\mathbf {x}}_{i}^{\prime }{\varvec{\beta }}) }{ \sum _{d \in {{{\mathcal {R}}}}_j} \exp ( {\mathbf {x}}_{d}^{\prime }{\varvec{\beta }})}, \end{aligned}$$
  the conditional probability that a particular unit *i* experiences the event at the *j*th survival time, given that exactly one unit in the risk set ${{{\mathcal {R}}}}_j$ experiences the event. The risk sets are not disjoint because a unit in ${{{\mathcal {R}}}}_j$ belongs to all risk sets for earlier events and the term “partial likelihood” is used (Cox, 1975).
(iii) Discrete choice: The conditional likelihood contribution for individual *j* in the conditional logit model for discrete choice takes the form of the standard partial likelihood contribution (15). Here, the likelihood contribution is the conditional probability that a particular alternative *i* is chosen by individual *j*, given that exactly one alternative is chosen from the individual-specific alternative set ${{{\mathcal {R}}}}_j$ (e.g., McFadden, 1974).

We also note that ${{{\mathcal {L}}}}_j^{\scriptscriptstyle \mathrm{CML}}$ is identical to the conditional likelihood contribution produced by instead conditioning on the order statistic $y_{(1)j},\ldots ,y_{(n_j)j}$ (e.g., Chen, 2007).

The basic idea of standard CML estimation is extended in *exact* conditional logistic regression (e.g., Cox, 1970; Mehta et al., 1995) with linear predictor (3). Here, each element in ${\varvec{\beta }}$ is estimated by conditioning on sufficient statistics for not just $\zeta _j$ (as in standard CML estimation) but also for the remaining elements of ${\varvec{\beta }}$. For small or unbalanced datasets this approach can mitigate separation problems where outcomes are perfectly predicted and standard conditional likelihoods therefore do not exist (e.g., Albert & Anderson, 1984). Moreover, inferences for ${\varvec{\beta }}$ are based on permutation distributions of the sufficient statistics that do not rely on asymptotics.

Nonparametric marginal maximum likelihood (NPMML) estimation (e.g., de Leuuw & Verhelst, 1986) leaves the latent variable distribution $p(\zeta _j)$ unspecified. NPMML estimation can be implemented by treating the latent variable as discrete and choosing the number of mass points to yield the highest likelihood. For concordant Rasch models that fit the observed sumscore distribution exactly, Lindsay et al. (1991) show that NPMML and CML estimation produce identical estimates of the item parameters ${\varvec{\beta }}$. Rice (2004) provides conditions ensuring that marginal and conditional likelihoods are equal. We note that standard NPMML estimation does not address the cluster-level endogeneity problem.

For a GLMM with linear predictor (1), the sufficient statistic for the latent variable vector ${\varvec{\zeta }}_j$ is $\sum _{i=1}^{n_j} {{\mathbf {z}}}_{ij}y_{ij}$. For the logit link and the Bernoulli distribution, the conditional likelihood contribution then takes the same form as (13), with the difference that $\sum _{i=1}^{n_j} {{\mathbf {z}}}_{ij}d_{i}=\sum _{i=1}^{n_j} {{\mathbf {z}}}_{ij}y_{ij}$ now replaces $\sum _{i=1}^{n_j} d_{i} = \sum _{i=1}^{n_j} y_{ij}$ in the definition of the permutation set. In a panel data setting, Thomas (2006) considered the special case of a logit model with a cluster-specific intercept and a cluster-specific slope of time.

### CML Estimation for Log Link and Poisson Distribution

Consider linear predictor (3) with a log link and a Poisson conditional distribution. Using that the sum of conditionally independent Poisson random variables has a Poisson distribution, the conditional likelihood contribution for cluster *j* becomes (e.g., Hausman et al., 1984)

$$\begin{aligned} {{{\mathcal {L}}}}_j^{\scriptscriptstyle \mathrm{CML}} \ = \ \frac{(\sum _{i=1}^{n_j} y_{ij})!}{\prod _{i=1}^{n_j} y_{ij}!} \, \prod _{i=1}^{n_j} \! \left( \frac{\exp ( {\mathbf {x}}_{ij}^{\prime }{\varvec{\beta }}) }{ \sum _{\ell =1}^{n_j}\exp ( {\mathbf {x}}_{\ell j}^{\prime }{\varvec{\beta }}) } \right) ^{y_{ij}}. \end{aligned}$$

Again $\zeta _j \! + \! {{\mathbf {v}}}_{j}^{\prime }{\varvec{\gamma }}$ has cancelled out of ${{{\mathcal {L}}}}_j^{\scriptscriptstyle \mathrm{CML}}$. We see that the product in ${{{\mathcal {L}}}}_j^{\scriptscriptstyle \mathrm{CML}}$ that contains ${\varvec{\beta }}$ is identical to the likelihood contribution for a unit *j* in a standard multinomial logit model with $n_j$ alternatives, except that it is not required that $y_{ij} \in \{0,1\}$ or $\sum _{i=1}^{n_j}y_{ij}=1$ here.

Recall that there is no incidental parameter problem for the model with log link and a Poisson conditional distribution, and CML estimation and JML estimation with dummy variables for clusters produce identical estimates of ${\varvec{\beta }}$. For the log link, assumption [A.2] is not required for consistent estimation and [A.4] can be relaxed by assuming that $\mu _{ij}$ is correctly specified (e.g., Wooldridge, 1999).

Thomas (2006) derived the CML estimator for a Poisson regression model with a cluster-specific intercept and a cluster-specific slope of time, and pointed out that there is no incidental parameter problem in this case either.

### CML Estimation Beyond GLM Link Functions

It is worth noting that CML estimation can be used not just for GLMMs with continuous, binary, and count outcomes that we have investigated so far but also for other combinations of outcomes and latent variable models.

- Binary $y_{ij} \in \{0,1\}$: Stratified linear odds-ratio models with cluster-specific parameters $\zeta _j$ (e.g., Storer et al., 1983)
  $$\begin{aligned} p(y_{ij}=1|{\mathbf {x}}_{ij},{{\mathbf {v}}}_{j};{{\varvec{\vartheta }}},\zeta _j) \ = \ \frac{\exp \{\zeta _j\} (1 + {\mathbf {x}}_{ij}^{\prime }{\varvec{\beta }}+ {{\mathbf {v}}}_j^{\prime }{\varvec{\gamma }})}{1 + \exp \{\zeta _j\} (1 + {\mathbf {x}}_{ij}^{\prime }{\varvec{\beta }}+ {{\mathbf {v}}}_j^{\prime }{\varvec{\gamma }})}. \end{aligned}$$
- Ordinal $y_{ij} \in \{0,\ldots , K\}$: Adjacent category logit models with cluster-specific parameters $\zeta _j$ (e.g., Heinen, 1996, p.124)
  $$\begin{aligned} p(y_{ij}=k|{\mathbf {x}}_{ij},{{\mathbf {v}}}_{j};{{\varvec{\vartheta }}},\zeta _j) \ = \ \frac{ \exp \{ \alpha _{ki} + {\mathbf {x}}_{ij}^{\prime }{\varvec{\beta }}+ {{\mathbf {v}}}_{j}^{\prime }{\varvec{\gamma }}+ k\zeta _j \} }{\sum _{c=0}^{K} \exp \{ \alpha _{ci} + {\mathbf {x}}_{ij}^{\prime }{\varvec{\beta }}+ {{\mathbf {v}}}_{j}^{\prime }{\varvec{\gamma }}+ c\zeta _j \} }, \end{aligned}$$
  where we explicitly let $\alpha _{ki}$ denote unit and category-specific intercepts (no intercepts in ${\varvec{\beta }}$ here), with $\alpha _{0i} \! = \! 0$. Seminal special cases in psychometrics include the partial credit model (Masters, 1982) that has no covariates, and the rating scale model (Andrich, 1978) where additionally the item parameters are decomposed as $\alpha _{ki}=\alpha _{i}+\kappa _{k}$. For the exchangeable case, $\alpha _{ki}$ is replaced by $\alpha _{k}$ with $\alpha _{0} \! = \! 0$. Approximate CML estimation can be obtained via data expansion for cumulative logit models (Mukherjee et al., 2008), of which the logistic graded response model of Samejima (1969) is a special case, and for continuation-ratio logit models, as shown for sequential item response models by Tutz (1990). Kelderman and Rijkes (1994) discuss CML estimation for a range of Rasch-type item response models for ordinal responses.
- Nominal $y_{ij} \in \{0,\ldots , K\}$: Multinomial logit models with cluster and category specific parameters $\zeta _{kj}$ (e.g., Chamberlain, 1980; Conaway, 1989; Lee, 2002)
  $$\begin{aligned} p(y_{ij}=k|{\mathbf {x}}_{0ij},\ldots ,{\mathbf {x}}_{Kij},{\mathbf {x}}_{ij},{{\mathbf {v}}}_j;{{\varvec{\vartheta }}},\zeta _{0j},\ldots ,\zeta _{Kj}) \ = \ \frac{ \exp \{\alpha _{ki} + {\mathbf {x}}_{kij}^{\prime }{\varvec{\beta }}+ {\mathbf {x}}_{ij}^{\prime }{\varvec{\beta }}_k + {{\mathbf {v}}}_{j}^{\prime }{\varvec{\gamma }}_k + \zeta _{kj}\} }{\sum _{c=0}^{K} \exp \{\alpha _{ci} + {\mathbf {x}}_{cij}^{\prime }{\varvec{\beta }}+ {\mathbf {x}}_{ij}^{\prime }{\varvec{\beta }}_c + {{\mathbf {v}}}_{j}^{\prime }{\varvec{\gamma }}_c + \zeta _{cj}\} }, \end{aligned}$$
  where $\alpha _{ki}$ are item and category-specific intercepts (no intercepts in ${\varvec{\beta }}$ or ${\varvec{\beta }}_k$ here), with $\alpha _{0i} \! = \! 0$ and $\alpha _{k1} \! = \! 0$, and we let ${\varvec{\beta }}_0 \! = \! {{\mathbf {0}}}$ and ${\varvec{\gamma }}_0 \! = \! {{\mathbf {0}}}$. Special cases in psychometrics (e.g., Rasch, 1961; Andersen, 1973b) do not include covariates. For the exchangeable case, $\alpha _{ki}$ is replaced by $\alpha _{k}$ with $\alpha _{0} \! = \! 0$.
- Survival times *t*: Stratified Cox-regression with cluster-specific baseline hazard function $h_{j}^{0}(t)$ (e.g., Chamberlain, 1985; Lancaster, 1990; Ridder & Tunali, 1999)
  $$\begin{aligned} h_{ij}(t) \ = \ h_{j}^{0}(t) \exp \{ {\mathbf {x}}_{ij}^{\prime }{\varvec{\beta }}+ {{\mathbf {v}}}_j^{\prime }{\varvec{\gamma }}\}, \end{aligned}$$
  where $h_{ij}(t)$ is the continuous time hazard function for unit *i* in cluster *j*. Estimating the models by maximum partial likelihood yields fixed-effects estimators in the spirit of CML estimation.

## MML Becomes CML for Large Clusters

Increasing the sample size usually does not improve estimation of misspecified models. However, standard MML estimation that ignores cluster-level endogeneity approaches CML estimation, and hence becomes more robust against cluster-level endogeneity, as the cluster sizes increase.

We first consider linear predictor (3) with an identity link. For known variance components, the generalized least squares (GLS) estimator is the MML estimator. Maddala (1971) showed that $\lim _{n \rightarrow \infty }{\widehat{{\varvec{\beta }}}}^{\scriptscriptstyle \mathrm GLS} = {\widehat{{\varvec{\beta }}}}^{\scriptscriptstyle \mathrm CML}$ for fixed *N*, see also (6) where $\omega \! \equiv \! \frac{\sigma ^2}{\sigma ^2+n\psi } \rightarrow 0$ when $n \rightarrow \infty$. Hence, GLS approaches CML estimation of ${\varvec{\beta }}$ as the cluster sizes increase, making GLS estimation robust against cluster-level endogeneity without any ameliorating model extensions. Moreover, ${\varvec{\gamma }}$ can in this case be consistently estimated if ${{\mathbf {v}}}_j$ is exogenous. It is clear from the IGLS formulae in Breusch (1987) that large-cluster robustness also applies to MML estimation.

Does the robustness extend beyond identity links? To shed some light on this, we performed a simulation study with exchangeable units to investigate the behaviour of MML estimation under cluster-level endogeneity for binary outcomes with a logit link and a normal latent variable distribution. For $N \! = \! 1,\!000,\!000$ clusters, we gradually increased the cluster sizes *n* from 2 to $1,\!000$. We simulated multivariate normal $(x_{1j},x_{2j},x_{3j},x_{4j},\zeta _j)$ with $\mathrm{Var}(x_{ij})=1$ and $\mathrm{Cor}(x_{ij},x_{i^{\prime }j})=0.2$ and parameter values $\gamma =0$, $\beta =1$, $\psi =1$.

**Fig. 3:**
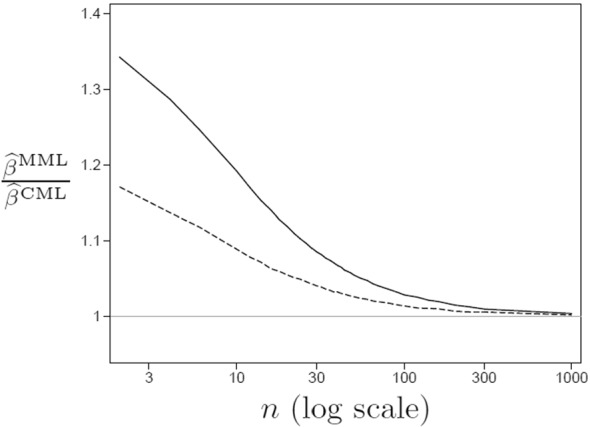
Automatic inconsistency correction of MML estimation for logistic random-intercept model as a function of cluster size *n*. $\mathrm{Cor}(\zeta _j,x_{ij}) = .4$ (solid curve) and $\mathrm{Cor}(\zeta _j,x_{ij}) = .2$ (dashed curve) for $N=1{,}000{,}000$ clusters.

In Fig. 3 we show the similarity of the MML estimator and the consistent (as $N \rightarrow \infty$) CML estimator by plotting $\frac{{\widehat{\beta }}^{\scriptscriptstyle \mathrm MML}}{{\widehat{\beta }}^{\scriptscriptstyle \mathrm CML}}$ against *n* for $\mathrm{Cor}(\zeta _j,x_{ij}) \! = \! 0.4$ (solid curve) and $\mathrm{Cor}(\zeta _j,x_{ij}) \! = \! 0.2$ (dashed curve). We see that the large-cluster robustness of MML estimation extends to the logit link and that larger cluster sizes are required to approach consistency when the cluster-level endogeneity is more severe.

## Extending the Scope of CML Estimation

For non-exchangeable data it is often plausible that the coefficients of the covariates ${\mathbf {x}}_{ij}$ and ${{\mathbf {v}}}_j$ are item-specific. Considering small to moderate cluster sizes, we now propose a useful extension of the model class for which CML estimation is applicable. Specifically, we generalize the GLMM in (1) by replacing ${\varvec{\beta }}$ and ${\varvec{\gamma }}$ by item-specific coefficients ${\varvec{\beta }}_i$ and ${\varvec{\gamma }}_i$

16 $$\begin{aligned} \nu _{ij} \ = \ {\mathbf {x}}_{ij}^{\prime }{\varvec{\beta }}_i + {{\mathbf {v}}}_{j}^{\prime }{\varvec{\gamma }}_{i} + {{\mathbf {z}}}_{ij}{\varvec{\zeta }}_{j}. \end{aligned}$$

Letting $n^{\mathrm{max}}$ denote the maximum cluster size, the linear predictor (16) can be re-expressed as

$$\begin{aligned} \nu _{ij} \ = \ \sum _{r=1}^{n^{\mathrm{max}}} ({d}_{ri}{\mathbf {x}}_{rj}^{\prime }){\varvec{\beta }}_r + \sum _{r=1}^{n^\mathrm{max}} (d_{ri}{{\mathbf {v}}}_{j}^{\prime }){\varvec{\gamma }}_{r} + {{\mathbf {z}}}_{ij}{\varvec{\zeta }}_{j}, \end{aligned}$$

where ${\mathbf {x}}_{rj}$ and ${{\mathbf {v}}}_{j}$ are now defined as in the exchangeable case. We see that this model includes solely unit-specific covariates $d_{ri}{\mathbf {x}}_{rj}$ and $d_{ri}{{\mathbf {v}}}_{j}$ and that both variable types have item-invariant coefficients. This is exactly the situation for GLMMs where CML estimation is traditionally employed, and CML estimation can therefore also be used for model (16) in a straightforward manner. Consistent estimation results for the regression coefficients in ${\varvec{\beta }}_i$ and the differences ${\varvec{\gamma }}_i - {\varvec{\gamma }}_{i^{\prime }}$ for $i^{\prime } \! \ne \! i$.

The validity of the traditional and rather restrictive model with invariant coefficients ${\varvec{\beta }}$ and ${\varvec{\gamma }}$ can now be investigated by contrasting it with the more general model (16), for instance by using *conditional* likelihood-ratio tests (Andersen, 1971) and fit measures based on conditional likelihoods. We refer to Maris (1998) for an insightful discussion of confidence intervals and hypothesis testing based on CML estimation.

CML estimation can also be used for models with crossed latent variables, such as panel models with both individual and year effects. For the identity link, Balazsi et al. (2017) review fixed-effects approaches, and for the logit link, Charbonneau (2017) and Kertesz (2017) use CML estimation by repeatedly conditioning on sufficient statistics to eliminate crossed latent variables one at a time. Generalized additive mixed models (GAMMs) with cluster-specific intercepts were estimated using CML by Zhang and Davidian (2004).

For models with several levels of nested latent variables, CML estimation is simply implemented by conditioning on the sufficient statistics for the latent variables at the lowest level.

## Mimicking CML by MML Estimation of Augmented Models

How can we proceed if CML estimation is not possible for the model of interest? A subset of the parameters can in some instances be treated as known to produce a model that lends itself to CML estimation. Verhelst and Glas (1995) proposed the one parameter logistic model (OPLM) where discrimination parameters are taken to be “fixed constants supplied by hypothesis”, making CML estimation feasible for ${\varvec{\beta }}$. However, we usually prefer approaches that can provide protective or mitigating estimation in general settings without treating parameters as known.

A model closely resembling that of interest can sometimes be found. The most obvious example is use of the logit link instead of the very similar probit link which cannot be used in CML estimation. Another example is dynamic (or autoregressive) logit models with cluster-specific intercepts for binary binary outcomes. In this case Bartolucci and Nigro (2010) used a quadratic exponential model (Cox & Wermuth, 1994) to address the limitations of the CML estimator of Honoré and Kyriazidou (2000).

In this section we consider a general approach where we mimick CML estimation by MML estimation of augmented models that can handle cluster-level endogeneity. The first variant uses an auxiliary model that specifies how the latent variable depends on the endogenous covariates and the second variant uses a joint model where the endogenous covariates are treated as outcomes. Both variants can accommodate multidimensional latent variables and, in contrast to CML, models with factor loadings or discrimination parameters and non-canonical link functions. For notational simplicity, we henceforth omit reference to parameters in all distributions.

### Auxiliary Modeling of $\zeta _j$ Given ${{\mathbf {w}}}_j$

Cluster-level endogeneity can be addressed by using an auxiliary statistical model for $p(\zeta _j|{{\mathbf {w}}}_j)$. We describe two alternative methods: using a GLLAMM and using a reduced-form GLMM.

#### Using GLLAMM

A GLLAMM (e.g., Rabe-Hesketh et al., 2004; Skrondal & Rabe-Hesketh, 2004) is composed of a response model for the outcomes given the covariates and latent variables and a structural model for the latent variables given the covariates. Conditional on latent variables, the response model is an extended GLM that accommodates more outcome types and different outcome types for different units. The linear predictor generalizes that of the GLMM in (1) by, for instance, allowing factor loadings or discrimination parameters for the latent variables. Conditional on observed covariates, the structural model is a multilevel structural equation model for the latent variables with normally distributed disturbance terms.

In the present setting, we can use a simple special case of a GLLAMM where the linear predictor of the response model is specified as (3) and the structural model is specified as the chosen auxiliary model. A Mundlak-inspired auxiliary model is

17 $$\begin{aligned} \zeta _j \ = \ {\overline{{\mathbf {x}}}}_{\cdot j}^{\prime }{{\varvec{\delta }}}+ u_j, \end{aligned}$$

and for non-exchangeable units we can use the more flexible Chamberlain auxiliary model (Chamberlain, 1980, 1984)

18 $$\begin{aligned} \zeta _j \ = \ \sum \nolimits {_{r=1}^{n_j}} \, {{\mathbf {x}}}_{r j}^{\prime } {{\varvec{\delta }}}_r + u_j. \end{aligned}$$

In (17) it is assumed that $\mathrm{E}(\zeta _j|{{\mathbf {w}}}_{j})= {\overline{{\mathbf {x}}}}_{\cdot j}^{\prime } {{\varvec{\delta }}}$, and $u_j$ is homoskedastic, normal and independent of ${\overline{{\mathbf {x}}}}_{\cdot j}$, and in (18) we assume that $\mathrm{E}(\zeta _j|{{\mathbf {w}}}_{j})= \sum _{r=1}^{n_j} {{\mathbf {x}}}_{r j}^{\prime } {{\varvec{\delta }}}_r$, and that $u_j$ is homoskedastic, normal and independent of the ${{\mathbf {x}}}_{ij}$ (Chamberlain, 1984).

Estimating the GLLAMM by MML provides consistent estimators for all parameters if the auxiliary and outcome models are correctly specified. MML estimation is mitigating for ${\varvec{\beta }}$ if the auxiliary model is a reasonable approximation of the correct model. Note that the assumed auxiliary models can be viewed as linear projections for the identity link (see Sect. 8.1.3).

Invoking the GLLAMM framework makes it straightforward to consider useful model extensions. For non-exchangeable units, it may be plausible that the observed covariates have item-specific coefficients ${\varvec{\beta }}_i$ and ${\varvec{\gamma }}_i$, and that the latent variable has item-specific factor loadings or discrimination parameters $\lambda _i$. We can then use the following linear predictor for the response model:

19 $$\begin{aligned} \nu _{ij} \ = \ {\mathbf {x}}_{ij}^{\prime }{\varvec{\beta }}_i + {{\mathbf {v}}}_{j}^{\prime }{\varvec{\gamma }}_i + \lambda _i\zeta _j. \end{aligned}$$

The model can, for instance, be extended to include multidimensional latent variables ${\varvec{\zeta }}_j$ and ultimately extended to the full GLLAMM response model (see e.g., Rabe-Hesketh et al., 2004).

#### Using Reduced-Form GLMM

Alternatively, the auxiliary model can be substituted into (3) to yield a reduced-form working GLMM. Substituting model (17), we obtain

$$\begin{aligned} g\{\mathrm{E}(y_{ij}|{\mathbf {x}}_{ij},{{\mathbf {v}}}_j,u_j)\} \ = \ {\mathbf {x}}_{ij}^{\prime }{\varvec{\beta }}+ {{\mathbf {v}}}_{j}^{\prime }{\varvec{\gamma }}+ {\overline{{\mathbf {x}}}}_{\cdot j}^{\prime }{{\varvec{\delta }}}+ u_j, \end{aligned}$$

which can be rearranged to get

20 $$\begin{aligned} g\{\mathrm{E}(y_{ij}|{\mathbf {x}}_{ij},{{\mathbf {v}}}_j,u_j)\} \ = \ ({\mathbf {x}}_{ij}-{\overline{{\mathbf {x}}}}_{\cdot j})^{\prime }{\varvec{\beta }}+ {\overline{{\mathbf {x}}}}_{\cdot j}^{\prime }({\varvec{\beta }}\! + \! {{\varvec{\delta }}}) + {{\mathbf {v}}}_{j}^{\prime }{\varvec{\gamma }}+ u_j. \end{aligned}$$

In general, MML estimation of these models produces mitigating estimation for ${\varvec{\beta }}$, with the exception of identity links where we have pointed out that the consistent CML estimator is obtained.

Neuhaus and McCulloch (2006) considered (20) without cluster-level covariates ${{\mathbf {v}}}_{j}$ and called it a “between-within model”. They referred to MML estimation of (20) as the “poor-man’s approximation to the conditional likelihood approach” because it is straightforward to implement in practice. Brumback et al. (2010) extended the poor-man’s approximation by considering nonlinear functions of the cluster-means. Note that, although ${\mathbf {X}}_j - {{\mathbf {1}}}_{n_j} \! \otimes {\overline{{\mathbf {x}}}}_{\cdot j}^{\prime }$ is orthogonal to ${{\mathbf {1}}}_{n_j} \! \otimes {{\mathbf {v}}}_{j}^{\prime }$, omitting ${{\mathbf {v}}}_{j}$ if ${\varvec{\gamma }}\! \ne \! {{\mathbf {0}}}$ is likely to produce some additional inconsistency for models with logit links because odds ratios are not collapsible (e.g., Gail et al., 1984).

For non-exchangeable units, we can substitute model (18) in (19) with $\lambda _i=1$ to obtain

$$\begin{aligned} g\{\mathrm{E}(y_{ij}|{\mathbf {x}}_{ij},{{\mathbf {v}}}_j,u_j)\} \ = \ {\mathbf {x}}_{ij}^{\prime }{\varvec{\beta }}_i + {{\mathbf {v}}}_{j}^{\prime }{\varvec{\gamma }}_i + \sum \nolimits {_{r=1}^{n_j}} {{\mathbf {x}}}_{r j}^{\prime } {{\varvec{\delta }}}_r + u_j. \end{aligned}$$

In contrast to the GLLAMM approach, factor loadings or discrimination parameters are not accommodated.

We conducted a Monte Carlo experiment to study the performance of MML estimation for a random-intercept binary logit model with a correctly specified auxiliary model for exchangeable data. To investigate consistency as $N\rightarrow \infty$ for $n=4$, we simulated multivariate normal $(x_{1j},x_{2j},x_{3j},x_{4j},\zeta _j)$ with $\mathrm{Var}(x_{ij})=1$, $\mathrm{Cor}(x_{ij},x_{i^{\prime }j})=0.2$ and $\mathrm{Cor}(x_{ij},\zeta _j)=0.4$ for all *i* and parameter values $\gamma =0$, $\beta =1$, and $\psi =1$. The cluster mean ${\overline{x}}_{\cdot j}$ was used in the auxiliary model.

**Fig. 4:**
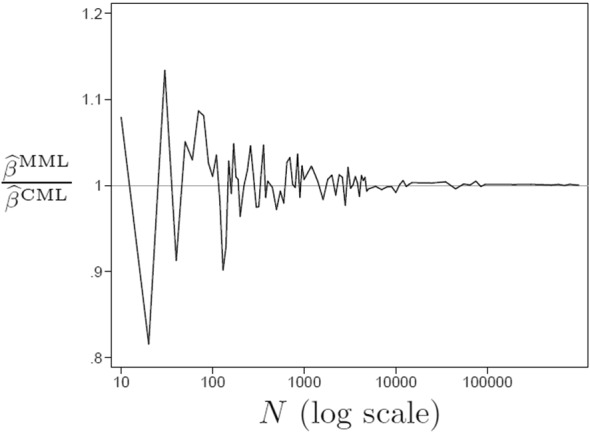
Protective MML estimate for simulated data with correct auxiliary model for logit link as a function of *N*. $\mathrm{Cor}(x_{ij},\zeta _j)=0.4$ and $n=4$.

Figure 4 plots the ratio of estimates $\frac{{\widehat{\beta }}^{\scriptscriptstyle \mathrm MML}}{{\widehat{\beta }}^{\scriptscriptstyle \mathrm CML}}$ against *N*. Because this ratio seems to converge to 1 as $N \rightarrow \infty$ for fixed *n* and we know that ${\widehat{{\varvec{\beta }}}}^{\scriptscriptstyle \mathrm CML}$ is consistent, we conclude that ${\widehat{{\varvec{\beta }}}}^{\scriptscriptstyle \mathrm MML}$ also appears to be consistent. MML estimation using a correct auxiliary model is therefore protective in this case.

### Joint Modeling of ${{\mathbf {y}}}_j$ and ${{\mathbf {w}}}_j$

We can also handle cluster-level endogeneity by specifying a joint statistical model $p({{\mathbf {y}}}_{j},{{\mathbf {w}}}_j)$ for the outcomes ${{\mathbf {y}}}_{j}$ and covariates ${{\mathbf {w}}}_j$. For continuous outcomes we discuss joint modeling via conventional structural equation modeling (SEM) and for other outcome types we briefly describe joint modeling using GLLAMMs.

#### Using Conventional SEM

In conventional SEM with identity links and normal conditional distributions, analytic integration over the latent variables is straightforward. In this case joint models are usually expressed as

$$\begin{aligned} p({{\mathbf {y}}}_{j},{{\mathbf {w}}}_j) \ = \ \int _{\zeta _j} p({{\mathbf {y}}}_{j}|{{\mathbf {w}}}_j,\zeta _j) {{p({{\mathbf {w}}}_j,\zeta _j)}} \, \mathrm{d} \zeta _j, \end{aligned}$$

which requires specification of a model for $p({{\mathbf {w}}}_j,\zeta _j)$. Specifically, a SEM that includes covariances between ${\mathbf {x}}_{ij}$ and $\zeta _j$ is specified and estimated by MML (e.g., Teachman et al., 2001; Bollen & Brand, 2010). In this case consistency requires a correctly specified covariance structure, but does not rely on normality (e.g., Browne, 1974), and the approach can be viewed as an instance of pseudo maximum (marginal) likelihood estimation (e.g., Arminger & Schoenberg, 1989).

**Fig. 5:**
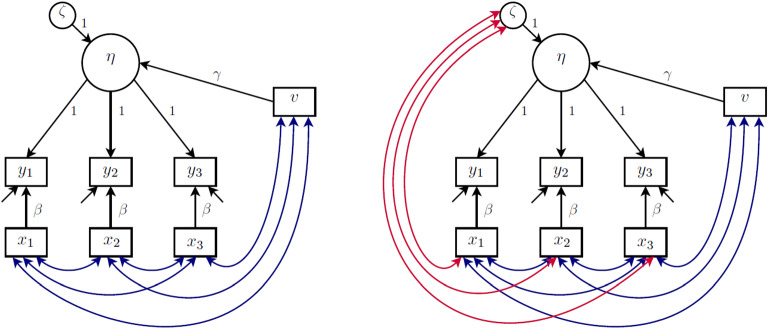
Joint modeling using SEM for identity link and normal conditional distribution. Path diagrams ($n=3$) for standard random-intercept model where $\mathrm{Cor}(x_{ij},\zeta _j) \! = \! 0$ and $\mathrm{Cor}(v_{j},\zeta _j) \! = \! 0$ (left panel) and joint SEM specifying $\mathrm{Cor}(x_{ij},\zeta _j) \! \ne \! 0$ and $\mathrm{Cor}(v_{j},\zeta _j) \! = \! 0$ (right panel).

Figure 5 shows path diagrams for a SEM representation of a standard random-intercept model with exogenous unit-specific covariate $x_{ij}$ and exogenous cluster-specific covariate $v_j$ (left panel) and a joint SEM for the same model but allowing the random intercept to be correlated with $x_{ij}$ to accommodate cluster-level endogeneity (right panel).

MML estimation of the joint model in the right panel produces CML estimates of ${\varvec{\beta }}$. In contrast to CML estimation, the joint MML approach is also consistent for ${\varvec{\gamma }}$ when ${{\mathbf {v}}}_j$ is cluster-level exogenous (as in the figure). Because all the bells and whistles of SEMs are available, it is straightforward to include, for instance, factor loadings in the models. Note that appropriate parameter restrictions should be imposed for exchangeable data (Sim, 2019).

#### Using GLLAMM

The SEM approach outlined above is not feasible beyond the identity link, and joint models in biometrics and statistics are typically formulated as (e.g., Neuhaus & McCulloch, 2006)

$$\begin{aligned} p({{\mathbf {y}}}_{j},{{\mathbf {w}}}_j) \ = \ \int _{\zeta _j} p({{\mathbf {y}}}_{j}|{{\mathbf {w}}}_j,\zeta _j) {{p({{\mathbf {w}}}_j|\zeta _j)}} p(\zeta _j) \, \mathrm{d} \zeta _j, \end{aligned}$$

which requires specification of a model for $p({{\mathbf {w}}}_j|\zeta _j)$. In this case a GLLAMM response model such as (19) with different outcome types for different units is specified for both the outcome model $p({{\mathbf {y}}}_j|{{\mathbf {w}}}_j,\zeta _j)$ and the covariate model $p({{\mathbf {w}}}_j|\zeta _j)$.

MML estimation for joint models is consistent for all model parameters if the model for $p({{\mathbf {w}}}_j|\zeta _j)$ is correctly specified, in addition to a correct outcome model for $p({{\mathbf {y}}}_{j}|{{\mathbf {w}}}_j,\zeta _j)$ and a correct $p(\zeta _j)$.

### Auxiliary or Joint Modeling?

Both auxiliary and joint modeling are useful for mimicking protective CML estimation of the target parameters ${\varvec{\beta }}$ when there is cluster-level endogeneity. However, it seems unlikely that auxiliary models represent plausible data-generating mechanisms (e.g., Goetgeluk & Vansteelandt, 2008), whereas joint models may do so, for instance when there is unobserved cluster-level confounding (see Sect. 12.1). In the unlikely event that the entire joint model is correctly specified, MML estimation will be consistent for *all* model parameters. Neuhaus and McCulloch (2006) discuss conditions for consistent estimation of ${\varvec{\beta }}$ using auxiliary modeling when the data-generating mechanism is a joint model. Auxiliary modeling can be implemented in standard GLMM software, and very flexible nonlinear parametric models can be used for $p(\zeta _j|{{\mathbf {w}}}_j)$, whereas modeling of $p({{\mathbf {w}}}_j|\zeta _j)$ requires specification of an appropriate link function and conditional distribution for each covariate. Joint modeling in effect assumes a particular dependence structure for the covariates but accommodates covariates missing at random. We advocate performing sensitivity analysis by using both auxiliary and joint modeling.

Finally, it should be kept in mind that the choice between CML estimation and MML estimation of augmented models may in practice involve a trade-off between inconsistencies due to CML estimation of overly simple models (e.g., without discrimination parameters) and misspecified endogeneity models.

## Reasons for Cluster-Level Endogeneity

Cluster-level endogeneity can arise for a variety of reasons, including unobserved cluster-level confounding, covariate measurement error, retrospective sampling, informative cluster sizes, missing data, and heteroskedasticity.

### Unobserved Cluster-Level Confounding of Causal Effects

**Fig. 6:**
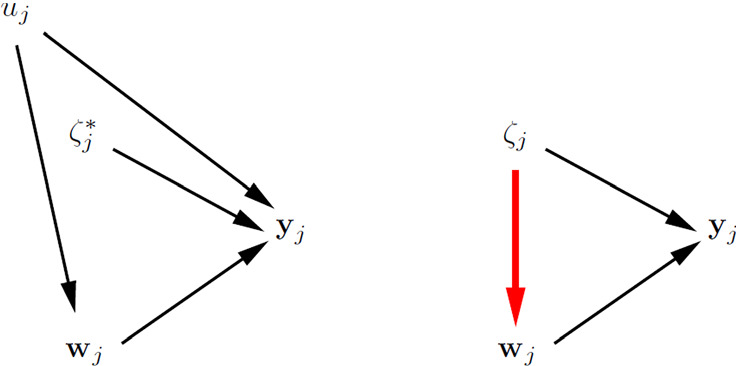
Unobserved cluster-level confounding. Cluster-level unobserved confounder $u_j$ (left panel) and resulting cluster-level endogeneity (right panel).

Recall that consistent MML estimation in general requires cluster-level exogeneity as shown in the left panel of Fig. 2. Consider now the case where the data-generating mechanism contains an unobserved cluster-level confounder $u_j$ as in the left panel of Fig. 6 (where the cluster-level error term is now denoted $\zeta _j^*$).

In a statistical model with linear predictor such as (3), the unobserved cluster-level confounder $u_j$ becomes absorbed by the cluster-level error term $\zeta _j = \zeta _j^* + u_j$ as displayed in the right panel of Fig. 6. It is evident that unobserved cluster-level confounding leads to cluster-level endogeneity.

Use of the term confounding presupposes that regression coefficients represent causal effects that can be confounded. Lancaster (2000, p. 296) points out that econometricians emphasize that some or all covariates may be “chosen” by an individual *j* in light of his knowledge of $\zeta _j$ (e.g., attending a training program, $x_{ij}\!=\!1$ rather than $x_{ij}\!=\!0$, because ability $\zeta _j$ is low). Hence, economic theory provides a presumption that $\zeta _j$ and ${\mathbf {x}}_{ij}$ are dependent in the population. Lancaster concludes that “This point plays absolutely no role in the statistics literature”, where $\zeta _j$ is invariably, and usually implicitly, either assumed to be independent or uncorrelated with random covariates or assumed to not depend on the values taken by fixed covariates. Here, regression coefficients merely represent associations between included variables, or linear projections in the case of linear models, in which case the error terms are orthogonal to the covariates by construction. Spanos (2006) contrasts the conventional meaning of models in econometrics and statistics.

Importantly, CML estimation can be consistent for causal effects even when there is unobserved cluster-level confounding. Under assumptions [A.1]-[A.4], ${\widehat{\beta }}^{\scriptscriptstyle \mathrm CML}$ for a treatment $x_{ij}$ in (3) can be interpreted as estimating a causal effect that is homogeneous in the population. However, causal effects are usually viewed as heterogeneous and the estimand taken to be some average causal effect (ACE) in the modern literature on causal inference.

For the identity link, ${\widehat{\beta }}^{\scriptscriptstyle \mathrm CML}$ represents an estimated ACE for the subpopulation of clusters where the treatment varies between the units (e.g., Imai & Kim, 2019; Petersen & Lange, 2020). Sobel (2012) and Wooldridge (2010: sect. 21.6.4) explore causal effects that can be estimated by fixed-effects methods for different treatment regimes and state assumptions required for identification. For the logit link, there is no simple interpretation of ${\widehat{\beta }}^{\scriptscriptstyle \mathrm CML}$ when the causal odds ratio is heterogeneous (Sjölander et al., 2012; Petersen & Lange, 2020).

### Cluster-Specific Measurement Error

Sometimes variables are fallibly measured with cluster-specific measurement errors (e.g., Wang et al., 2012). Examples include teacher-specific bias in ratings of students and laboratory tests analyzed in batches.

#### Covariate Measurement Error

We now consider the following version of linear predictor (3):

21 $$\begin{aligned} \nu _{ij} \ = \ \beta x_{ij} + {{\mathbf {v}}}_{j}^{\prime }{\varvec{\gamma }}+ \zeta _j, \end{aligned}$$

where the unit-specific covariate $x_{ij}$ is continuous.

If $x_{ij}$ were observed and cluster-level exogenous, consistent estimation of all model parameters could proceed by MML estimation. The new feature is that $x_{ij}$ is latent and fallibly measured by a continuous variable $m_{ij}$ with additive cluster-specific covariate measurement error $\delta _{j}$

$$\begin{aligned} m_{ij} \ = \ x_{ij} + \delta _{j}. \end{aligned}$$

Rearranging this classical covariate measurement model as $x_{ij} = m_{ij} - \delta _{j}$, we substitute it in (21) to obtain a working model with linear predictor

$$\begin{aligned} \nu _{ij} \ = \ \beta m_{ij} + {{\mathbf {v}}}_{j}^{\prime }{\varvec{\gamma }}+ \zeta _j^*, \end{aligned}$$

where $\zeta _j^* \equiv \zeta _j - \beta \delta _{j}$. Having replaced the latent covariate $x_{ij}$ by the fallibly observed covariate $m_{ij}$, we see that $\zeta _j$ has been replaced by a composite cluster-specific error term. Unfortunately, even if $x_{ij}$ is cluster-level exogenous, the fallibly observed covariate $m_{ij}$ becomes cluster-level endogenous. This is because the component $\beta \delta _{j}$ of $\zeta _j^*$ is not independent of $m_{ij}$.

MML estimation would be inconsistent for $\beta$ in the working model. Joint modeling of the outcomes $y_{ij}$ and measures $m_{ij}$ would enable consistent MML estimation of all model parameters, but only if the entire model is correctly specified. In contrast, CML estimation is protective for $\beta$. In this case parametric assumptions are not required for the distributions of $\zeta _j$ or $\delta _{j}$, and these terms could even be dependent, producing a form of differential measurement error. Moreover, $x_{ij}$ could be cluster-level endogenous. CML estimation remains protective if several continuous unit-specific covariates are measured with covariate- and cluster-specific errors.

#### Latent-Response Measurement Error

Consider now the class of GLMMs that can be expressed as latent response models

22 $$\begin{aligned} y_{ij}^* \ = \ {\mathbf {x}}_{ij}^{\prime }{\varvec{\beta }}+ {{\mathbf {v}}}_{j}^{\prime }{\varvec{\gamma }}+ \zeta _j + \epsilon _{ij}. \end{aligned}$$

For instance, models with logit links for binary outcomes $y_{ij}$ arise if $\epsilon _{ij}$ has a standard logistic density and the observed outcome is produced by thresholding the latent response $y_{ij}^*$ as $y_{ij} = \text{ I }({y_{ij}^*>0})$.

If $y_{ij}^*$ is contaminated by cluster-specific additive error $\delta _j$, we obtain $y_{ij}^{\bullet } = y_{ij}^* + \delta _j$ yielding observed outcomes $y_{ij} = \text{ I }({y_{ij}^{\bullet }>0})$. Substituting the latent response model (22) we see that $\zeta _j$ is replaced by a composite cluster-specific intercept $\zeta _j^{\bullet } = \zeta _j + \delta _j$.

Again, CML estimation is protective for $\beta$ and parametric assumptions are not required for $\zeta _j$ and $\delta _{j}$. Moreover, differential measurement error, in the sense that $\delta _j$ depends on ${\mathbf {x}}_{ij}$, ${{\mathbf {v}}}_{j}$ and $\zeta _j$, is accommodated.

### Retrospective Sampling

We will discuss two kinds of retrospective sampling schemes that produce cluster-level endogeneity, the first by sampling units and the second by sampling clusters.

#### Case-Control and Choice-Based Sampling of Units

Case-control sampling is very useful for rare binary outcomes ${{\mathbf {y}}}_j$ when obtaining one or more of the covariates is expensive or invasive (e.g., Breslow, 1996). Examples include drawing blood samples from individuals and conducting comprehensive psychiatric interviews with patients.

The basic idea of case-control designs is to under-sample units *i* with outcome $y_{ij}=0$. This is an example of retrospective sampling because the probability of being sampled depends on the value taken by an outcome variable. Letting $S_{ij}$ be an indicator variable for sampling unit *i* in cluster *j*, the probability of selecting the unit is then dependent on whether the unit is a case ($y_{ij}=1$) or control ($y_{ij}=0$):

$$\begin{aligned} p(S_{ij} \!=\! 1|y_{ij}) \equiv \pi (y_{ij}). \end{aligned}$$

We assemble the selection indicators for the units in cluster *j* in the selection vector ${{\mathbf {s}}}_j$. In a cumulative case-control study (e.g., Rothman et al., 2008, p. 125), the researcher samples all cases, $\pi (1) = 1$, whereas $\pi (0)$ is small in order to under-sample controls.

In choice-based sampling individuals are sampled retrospectively by stratifying on their individual choices (e.g., Manski, 1981), in which case the marginal distribution of the choices in the selected sample typically differs from the corresponding population distribution. A canonical example is choice of transport mode (such as bus, plane or train) where travelers are interviewed at their chosen mode.

**Fig. 7:**
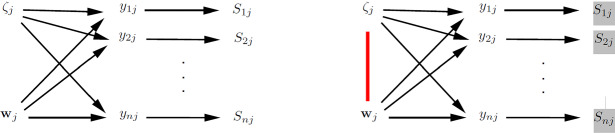
Retrospective sampling of units. Unselected population (left panel) and selected sample (right panel).

The anatomy of retrospective sampling of units is depicted in the left panel of Fig. 7, where we see that the probability of selecting a unit depends on the outcome of that particular unit. Importantly, the outcomes ${{\mathbf {y}}}_{j}$ are colliders because they are affected by both $\zeta _j$ and ${{\mathbf {w}}}_j$. The elements of ${{\mathbf {s}}}_j$ are descendants of the colliders in ${{\mathbf {y}}}_j$ and conditioning on ${{\mathbf {s}}}_j$ (performing selection) therefore induces cluster-level endogeneity as illustrated in the right panel of Fig. 7 (conditioning is henceforth signalled by placing variables in grey background in the figures). The produced dependence between the “parents” $\zeta _j$ and ${{\mathbf {w}}}_j$ is due to “moralization” according to the terminology of Lauritzen et al. (1990).

Applications proceed with the logit link

23 $$\begin{aligned} \text{ logit }\{p(y_{ij}\!=\!1|{\mathbf {x}}_{ij},{{\mathbf {v}}}_j,\zeta _j)\} \ = \ \alpha _i + {\mathbf {x}}_{ij}^{\prime }{\varvec{\beta }}+ {{\mathbf {v}}}_{j}^{\prime }{\varvec{\gamma }}+ \zeta _j, \end{aligned}$$

where we temporarily denote the intercept for unit *i* as $\alpha _i$ (no intercepts in ${\varvec{\beta }}$ here).

The model is often estimated by standard MML but the marginal likelihood contribution is misspecified for the selected sample (where we denote the outcome vector as ${{\mathbf {y}}}_{j}^{\scriptscriptstyle \mathrm sel}$)

$$\begin{aligned} p({{\mathbf {y}}}_{j}^{\scriptscriptstyle \mathrm sel}|{{\mathbf {s}}}_j,{{\mathbf {w}}}_j)= & {} \int _{\zeta _j} \left\{ \prod _{i} {p(y_{ij}^{\scriptscriptstyle \mathrm sel}|S_{ij}\!=\!1,{\mathbf {x}}_{ij},{{\mathbf {v}}}_j,\zeta _j)} \right\} {p(\zeta _j|{{\mathbf {s}}}_j,{{\mathbf {w}}}_j)} \, \mathrm{d} \zeta _j \\\ne & {} \int _{\zeta _j} \left\{ \prod _{i} p(y_{ij}^{\scriptscriptstyle \mathrm sel}|{\mathbf {x}}_{ij},{{\mathbf {v}}}_j,\zeta _j) \right\} {p(\zeta _j)} \, \mathrm{d} \zeta _j \\ \end{aligned}$$

We see that the correct contribution in the first line differs from the standard marginal likelihood contribution in the second line in two ways: (1) the correct conditional outcome distribution $p(y_{ij}^{\scriptscriptstyle \mathrm sel}|S_{ij}\!=\!1,{\mathbf {x}}_{ij},{{\mathbf {v}}}_j,\zeta _j)$ differs from the naive one $p(y_{ij}^{\scriptscriptstyle \mathrm sel}|{\mathbf {x}}_{ij},{{\mathbf {v}}}_j,\zeta _j)$ and the correct latent variable distribution $p(\zeta _j|{{\mathbf {s}}}_j,{{\mathbf {w}}}_j)$ differs from the naive one $p(\zeta _j)$. The fact that the correct latent variable distribution becomes $p(\zeta _j|{{\mathbf {s}}}_j,{{\mathbf {w}}}_j)$ corresponds to the dependence between $\zeta _j$ and ${{\mathbf {w}}}_j$ seen in the right panel of Fig. 7. As a result, standard MML estimation leads to inconsistent estimation of ${\varvec{\beta }}$, an instance of collider-stratification bias (e.g., Greenland et al., 1999).

It is important to note that the logit link is preserved in selected samples

$$\begin{aligned} \text{ logit }\{p(y_{ij}^{\scriptscriptstyle \mathrm sel}\!=\!1|S_{ij}\!=\!1,{\mathbf {x}}_{ij},{{\mathbf {v}}}_j,\zeta _j)\} \ = \ \alpha _i^{\scriptscriptstyle \mathrm sel} + {\mathbf {x}}_{ij}^{\prime }{\varvec{\beta }}+ {{\mathbf {v}}}_{j}^{\prime }{\varvec{\gamma }}+ \zeta _j, \end{aligned}$$

where $\alpha _i^{\scriptscriptstyle \mathrm sel} = \alpha _i + \log [\pi (1)/\pi (0)]$. It follows that standard CML estimation is protective. Prentice (1976) argues that protective estimation of the coefficient $\beta$ for a binary $x_{ij}$ can alternatively be achieved by CML estimation of a *retrospective* logit model with cluster-specific effects where $x_{ij}$ is the outcome and $\beta$ is now the coefficient of $y_{ij}$. In contrast, MML estimation with an auxiliary model produces inconsistent estimation of the intercepts but protective or mitigating estimation of the coefficients of covariates, depending on whether the auxiliary model is correct or not.

#### Sumscore-Based Sampling of Clusters

For rare binary outcomes ${{\mathbf {y}}}_j$, clusters where many of the outcomes take the value zero are sometimes under-sampled. For instance, in a genetic study a family *j* could be more likely to be ascertained if one or more members of the family has a particular disease.

Here we consider the case where the probability of sampling a cluster *j* is a function of the sumscore or number of “successes” in the cluster (e.g., Neuhaus & Jewell, 1990)

$$\begin{aligned} p(S_j \!=\! 1|{{\mathbf {y}}}_j) \ = \ f \left( \mathop {\Sigma }\limits _{i=1}^{n_j} y_{ij}\right) . \end{aligned}$$

**Fig. 8:**
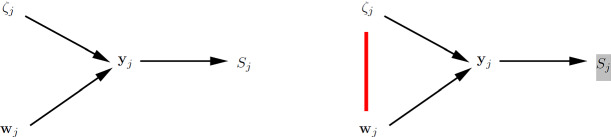
Retrospective sampling of clusters. Unselected population (left panel) and selected sample (right panel).

The structure of retrospective sumscore-based sampling is shown in the left panel of Fig. 8. Importantly, we see from the right panel that conditioning on the cluster-selection indicator $S_j$ induces cluster-level endogeneity because $S_j$ is a descendant of the colliders in ${{\mathbf {y}}}_j$.

We proceed with a logit link as we did for retrospective sampling of units. The standard marginal likelihood contribution is now misspecified in the selected sample

$$\begin{aligned} p({{\mathbf {y}}}_{j}^{\scriptscriptstyle \mathrm sel}|S_j\!=\!1,{{\mathbf {w}}}_j)= & {} \int _{\zeta _j} {p({{\mathbf {y}}}_{j}^{\scriptscriptstyle \mathrm sel}|S_j\!=\!1,{\mathbf {x}}_{ij},{{\mathbf {v}}}_j,\zeta _j)} {p(\zeta _j|S_j \!=\! 1,{{\mathbf {w}}}_j)} \, \mathrm{d} \zeta _j \\\ne & {} \int _{\zeta _j} \left\{ \prod _{i=1}^{n_j} p(y_{ij}^{\scriptscriptstyle \mathrm sel}|{\mathbf {x}}_{ij},{{\mathbf {v}}}_j,\zeta _j) \right\} {p(\zeta _j)} \, \mathrm{d} \zeta _j, \end{aligned}$$

which yields inconsistent MML estimation of ${\varvec{\beta }}$. In contrast to retrospective sampling of units, $p({{\mathbf {y}}}_{j}^{\scriptscriptstyle \mathrm sel}|S_j\!=\!1,{\mathbf {x}}_{ij},{{\mathbf {v}}}_j,\zeta _j)$ is no longer a product of conditionally independent logit models, but rather corresponds to the Rosner (1984) model for correlated data. MML estimation based on an auxiliary model therefore produces inconsistent estimation of ${\varvec{\beta }}$. Fortunately, standard CML estimation is once again protective for ${\varvec{\beta }}$ (Neuhaus & Jewell, 1990).

### Informative Cluster Sizes

**Fig. 9:**
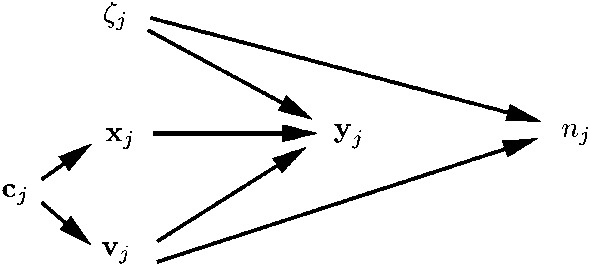
Informative cluster-sizes.

It is sometimes plausible that the cluster sizes $n_j$ depend on a cluster-specific latent variable $\zeta _j$ and cluster-specific covariates ${{\mathbf {v}}}_j$. For example, for a clinical psychologist *j*, the patient volume $n_j$ may depend on his latent skill $\zeta _j$ and whether he works in a public or private hospital $z_j$. The patient outcomes ${{\mathbf {y}}}_j$ may depend on $\zeta _j$, $z_j$, and observed individual patient characteristics ${\mathbf {x}}_j$. This situation is shown in Fig. 9, where ${{\mathbf {c}}}_j$ is included to signify that ${\mathbf {x}}_j$ and ${{\mathbf {v}}}_j$ are typically dependent.

A joint model can be specified for the outcomes ${{\mathbf {y}}}_j$ and cluster size $n_j$, the latter part typically a Poisson model with log link

$$\begin{aligned} p({{\mathbf {y}}}_{j},n_j|{\mathbf {x}}_j,{{\mathbf {v}}}_j) \ = \ \int _{\zeta _j} p(n_j|{{\mathbf {v}}}_j,\zeta _j) \left\{ \prod _{i=1}^{n_j} p(y_{ij}|{\mathbf {x}}_{ij},{{\mathbf {v}}}_j,\zeta _j) \right\} p(\zeta _j) \, \mathrm{d} \zeta _j. \end{aligned}$$

MML estimation of the joint model is consistent if the cluster-size model $p(n_j|{{\mathbf {v}}}_j,\zeta _j)$ is correctly specified in addition to the outcome model $p(y_{ij}|{\mathbf {x}}_{ij},{{\mathbf {v}}}_j,\zeta _j)$ and the mixing distribution $p(\zeta _j)$.

Researchers occasionally condition on $n_j$ by including it as a covariate in the model (e.g., Seaman et al., 2014). The standard marginal likelihood contribution in this case is misspecified

$$\begin{aligned} p({{\mathbf {y}}}_{j}|{\mathbf {x}}_j,{{\mathbf {v}}}_j,n_j)= & {} \int _{\zeta _j} \left\{ \prod _{i=1}^{n_j} p(y_{ij}|{\mathbf {x}}_{ij},{{\mathbf {v}}}_j,\zeta _j,n_j) \right\} {p(\zeta _j|{\mathbf {x}}_j,{{\mathbf {v}}}_j,n_j)} \, \mathrm{d} \zeta _j \\= & {} \int _{\zeta _j} \left\{ \prod _{i=1}^{n_j} p(y_{ij}|{\mathbf {x}}_{ij},{{\mathbf {v}}}_j,\zeta _j,n_j) \right\} {p(\zeta _j|{{\mathbf {v}}}_j,n_j)} \, \mathrm{d} \zeta _j \\\ne & {} \int _{\zeta _j} \left\{ \prod _{i=1}^{n_j} p(y_{ij}|{\mathbf {x}}_{ij},{{\mathbf {v}}}_j,\zeta _j,n_j) \right\} p(\zeta _j) \, \mathrm{d} \zeta _j. \end{aligned}$$

It is evident from Fig. 9 that $n_j$ is a collider and that conditioning on $n_j$ opens a confounding path between $\zeta _j$ and ${\mathbf {x}}_j$ that makes ${\mathbf {x}}_j$ cluster-level endogenous. However, also conditioning on ${{\mathbf {v}}}_j$ blocks this path, in which case ${\mathbf {x}}_j$ remains cluster-level exogenous, whereas ${{\mathbf {v}}}_j$ becomes cluster-level endogenous. The latent-variable distribution becomes $p(\zeta _j|{{\mathbf {v}}}_j,n_j)$ (shown in the second line of the likelihood contribution above), which differs from the assumed $p(\zeta _j)$ in naive MML estimation.

For the identity link, MML estimation is consistent for ${\varvec{\beta }}$ under cluster-level exogeneity whatever the distribution of $\zeta _j$ (e.g., Verbeke & Lesaffre, 1997), but this is not the case for other links. Naive MML estimation that includes $n_j$ as a covariate therefore gives protective estimation of ${\varvec{\beta }}$ for the identity link, but otherwise inconsistent estimation, albeit mildly inconsistent according to simulations. Fortunately, standard CML estimation is protective for ${\varvec{\beta }}$ for all canonical links because $n_j$ is a cluster-level characteristic. Important advantages of this approach are that we neither need to know about the relevant ${{\mathbf {v}}}_j$ nor specify a correct model for the dependence on $n_j$.

In practice, the cluster size $n_j$ is often ignored in the estimating model. From Fig. 9 we see that conditioning on ${{\mathbf {v}}}_j$ makes $n_j$ and ${\mathbf {x}}_j$ conditionally independent. This implies that MML estimation for the identity link remains protective for ${\varvec{\beta }}$. For the logit link, the estimator of ${\varvec{\beta }}$ for the model that ignores $n_j$ has a probability limit that differs from the inconsistent MML estimator for the model where $n_j$ is included as a covariate because odds-ratios are not collapsible (e.g., Gail et al., 1984). It is extremely unlikely that this estimator is consistent. For the log link, the inconsistency is expected to be mild. Fortunately, standard CML estimation remains protective for ${\varvec{\beta }}$ for all canonical links.

Neuhaus and McCulloch (2011) considered a restrictive version of the model in Fig. 9 where the cluster size just depends on $\zeta _j$. Here, there is no collider problem and the correct mixing distribution becomes $p(\zeta _j|n_j)$. The conclusions reported above for MML and CML estimation persist.

### Data Missing Not at Random

#### Outcome-Dependent Missingness

Missingness of outcomes that depends on the values taken by the outcomes violates the missing at random assumption (e.g., Seaman et al., 2013) and is therefore referred to as not missing at random (NMAR).

**Fig. 10:**
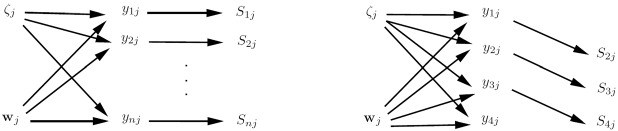
Outcome dependent missingness. Current outcome dependent missingness (left panel) and lag(1) dependent missingness for $n=4$ (right panel).

In the longitudinal non-exchangeable setting, current-outcome dependent missingness occurs if the probability that an outcome is missing at an occasion *i*, $S_{ij}=1$, depends on the value taken by the outcome for that particular occasion $y_{ij}$. An example would be when the outcome is a disease symptom that makes it more difficult to visit a clinic for an assessment. The structure of current-outcome dependent missingness is shown in the left panel of Fig. 10, which is identical to that previously shown for retrospective sampling of units in Fig. 7.

Conditioning on the selection indicators $S_{ij}$ leads to cluster-level endogeneity, as shown in the right panel of Fig. 7, because the $S_{ij}$ are decendants of the colliders $y_{ij}$. For the non-exchangeable case, it is plausible that the missingness probabilities will differ between units *i*, $\pi _i(y_{ij})$.

For exchangable units, outcome-dependent missingness only makes sense if the probability that an outcome $y_{ij}$ is missing for a unit, $S_{ij}=1$, depends on the value taken by the outcome for that particular unit. In contrast, for longitudinal data we can also consider lag(1) outcome-dependent missingness where the probability that an outcome is missing at an occasion, $S_{ij}=1$, depends on the previous outcome $y_{i-1,j}$. Such a process is shown in the right panel of Fig. 10 for the case of $n=4$. This can occur if an outcome, such as a diagnosis, only affects missingness after having been relayed to the subject. Again, missingness produces cluster-level endogeneity.

Hausman and Wise (1979) and Diggle and Kenward (1994) used MML to estimate joint models where linear predictor (3) with an identity link for the outcome is combined with probit/logit models for current-outcome dependent and current plus lag(1) outcome-dependent missingness, respectively. MML estimation is consistent for all parameters under correct model specification, but this approach has been criticized for relying heavily on unverifiable distributional assumptions (e.g., Little, 1985). Standard MML estimation (ignoring the missingness) suffers from collider-stratification bias and is inconsistent for ${\varvec{\beta }}$.

Skrondal and Rabe-Hesketh (2014) obtain several useful results for model (23). For current outcome-dependent missingness, CML estimation is protective for ${\varvec{\beta }}$. MML estimation using an auxiliary model yields protective estimation of ${\varvec{\beta }}$ if that model is correct and mitigating estimation otherwise. For lag(1) outcome-dependent missingness, CML estimation stratified on the missingness pattern is protective. If missingness depends on both the current and all lagged outcomes (whether observed or missing), CML estimation is protective only if complete data are analyzed stratified on judiciously chosen values of the sufficient statistic. Note that none of these results hinge on specification of a parametric or nonparametric model for missingness.

#### Latent-Variable and Covariate Dependent Missingness

Missingness of outcomes can depend on the latent variable in addition to the covariates and is in this case also not missing at random (NMAR). For example, the probability of visiting a clinic/answering an item could depend on the unobserved frailty/ability $\zeta _j$ of the subject as well as his observed characteristics ${{\mathbf {w}}}_j$.

**Fig. 11:**
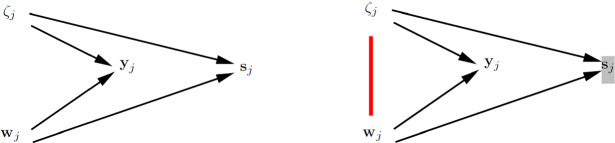
Latent-variable and covariate dependent missingness. Unselected population (left panel) and selected sample (right panel).

The structure shown in Fig. 11 is similar to that previously discussed for informative cluster-sizes but here ${{\mathbf {s}}}_{j}$ could also depend on ${\mathbf {x}}_{j}$, so conditioning on ${{\mathbf {v}}}_j$ does not block the confounding path to give protective estimation of ${\varvec{\beta }}$.

Joint modeling can be performed with “shared-parameter” models (e.g., Wu & Carroll, 1988; Ten Have et al., 1998) where the outcome and missingness processes share latent variables in addition to observed covariates. MML estimation is consistent for all parameters if the joint model is correctly specified, but note that such models rely on unverifiable distributional assumptions (e.g., Little, 1985). Standard MML estimation that ignores missingness suffers from collider-stratification bias and is inconsistent. CML estimation is protective for ${\varvec{\beta }}$ for identity and log link functions because it can be cast as JML estimation of models with cluster-specific dummy variables.

For the logit link, Skrondal and Rabe-Hesketh (2014) prove that CML estimation is protective for ${\varvec{\beta }}$, even when missingness also depends on missing outcomes (in addition to the latent variable and covariates). Again, this does not require specification of a parametric or nonparametric missingness model.

**Fig. 12:**
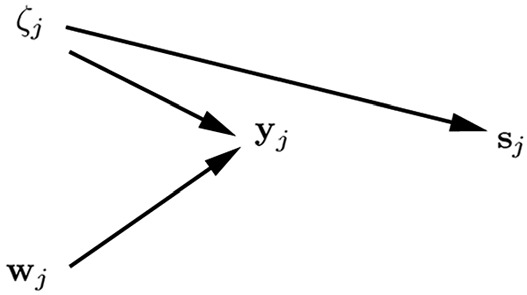
Latent-variable dependent missingness.

The case where missingness just depends on the latent variable is shown in Fig. 12. Here the shape of the latent variable distribution is changed from $p(\zeta _j)$ to $p(\zeta _j|{{\mathbf {s}}}_j)$ but there is no collider problem. For the identity link, standard MML estimation is now protective for ${\varvec{\beta }}$. In contrast, standard MML estimation of ${\varvec{\beta }}$ for the logit link is just mitigating, but with mild inconsistency (e.g., Neuhaus et al., 1992). CML estimation remains protective for ${\varvec{\beta }}$.

### Heteroskedastic Latent Variable

**Fig. 13:**
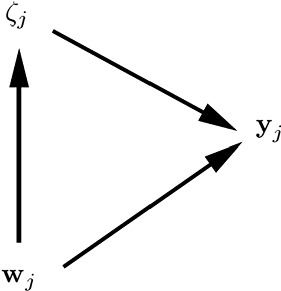
Heteroskedastic latent variable.

Cluster-level endogeneity occurs if the variance of the latent variable distribution depends on cluster-level covariates ${{\mathbf {v}}}_j$. For example, Heagerty (1999) considered a longitudinal study of schizophrenia where the latent variable variance depends on gender. Such heteroskedasticity is illustrated in Fig. 13 where ${{\mathbf {v}}}_j$ is a subset of ${{\mathbf {w}}}_j$.

For the identity link, standard MML estimation remains consistent under this misspecification. For the logit link, Heagerty and Kurland (2001) demonstrated that standard MML estimation becomes inconsistent for all parameters. If the structure of the heteroskedasticity is known, consistent MML estimation can be achieved if an appropriate heteroskedasticity model can be specified, for instance by using a GLLAMM. Fortunately, standard CML estimation is protective for ${\varvec{\beta }}$, and does not require additional modeling or even knowing the variables that induce heteroskedasticity.

## Latent Variable Scoring

When considering a latent variable model without an incidental parameters problem, it is straightforward to obtain estimates of $\zeta _j$ by JML estimation using (4). In general, this scoring method can work well for large cluster sizes $n_j$.

CML estimation is of limited value if the target of inference is the value of the latent variable $\zeta _j$. However, we can estimate the cluster-specific component $u_j \equiv \zeta _j + h({{\mathbf {v}}}_j)$ that is eliminated in CML estimation by maximizing the scoring likelihood

$$\begin{aligned} \prod _{i=1}^{n_j} p(y_{ij}|{\mathbf {x}}_{ij}; {\widehat{{{\varvec{\vartheta }}}}}^{\mathrm{{\scriptscriptstyle CML}}}, u_j) \end{aligned}$$

with respect to $u_j$. The estimated scores ${\widehat{u}}_j^{\mathrm{{\scriptscriptstyle ML}}}$ can then be plugged in to obtain predictions of outcomes $p(y_{ij}|{\mathbf {x}}_{ij}; {\widehat{{{\varvec{\vartheta }}}}}^{\mathrm{{\scriptscriptstyle CML}}}, {\widehat{u}}_j^{\mathrm{{\scriptscriptstyle ML}}})$. Improvements of the ML estimator, such as variants of the weighted likelihood method of Warm (1989), can also be employed.

If CML estimation is mimicked by MML estimation of augmented models, we can use empirical Bayes (EB) prediction that performs partial pooling of information from other clusters and is therefore more precise. For instance, for joint modeling (see Sect. 11.2) the predictions can be obtained as:

$$\begin{aligned} {\widetilde{\zeta }}_j \ = \ \frac{\int _{\zeta _j} \zeta _j \, p({{\mathbf {y}}}_{j}|{{\mathbf {w}}}_j,\zeta _j; {\widehat{{{\varvec{\vartheta }}}}}^{\scriptscriptstyle \mathrm MML}) {{p({{\mathbf {w}}}_j|\zeta _j; {\widehat{{{\varvec{\vartheta }}}}}^{\scriptscriptstyle \mathrm MML})}} \phi (\zeta _j;0,{\widehat{\psi }}^{\scriptscriptstyle \mathrm MML}) \, \mathrm{d} \zeta _j}{\int _{\zeta _j} p({{\mathbf {y}}}_{j}|{{\mathbf {w}}}_j,\zeta _j; {\widehat{{{\varvec{\vartheta }}}}}^{\scriptscriptstyle \mathrm MML}) {{p({{\mathbf {w}}}_j|\zeta _j; {\widehat{{{\varvec{\vartheta }}}}}^{\scriptscriptstyle \mathrm MML})}} \phi (\zeta _j;0,{\widehat{\psi }}^{\scriptscriptstyle \mathrm MML}) \, \mathrm{d} \zeta _j}. \end{aligned}$$

The performance of EB prediction in this case also relies on correct specification of $p({{\mathbf {w}}}_j|\zeta _j; {{\varvec{\vartheta }}})$. Parametric assumptions are moreover made regarding the latent variable distribution unless nonparametric marginal maximum likelihood ((NPMML) is used (Rabe-Hesketh et al., 2003). However, simulation studies suggest that violations of the distributional assumptions may have a modest impact on the mean squared error of prediction unless the assumed distribution has more limited support than the correct distribution, the latent variable variance is large, or the cluster sizes are large (e.g., McCulloch & Neuhaus, 2011a; b).

We refer to Skrondal and Rabe-Hesketh (2009) for latent variable scoring and various kinds of prediction of outcomes in GLMMs and related models with multidimensional latent variables.

## Concluding Remarks

We have demonstrated that conditional likelihoods have an important role to play in latent variable modeling that extends well beyond Rasch models for measurement. For the class of models considered here, a great advantage of CML estimation is that it can *simultaneously* handle cluster-level endogeneity problems induced by, for instance, unobserved cluster-level confounding of causal effects, cluster-specific measurement error, retrospective sampling, informative cluster sizes, missing data, and heteroskedasticity.

Although randomized experimental designs ensure that there is no confounding of treatment effects, there could be cluster-level endogeneity due to, for instance, covariate measurement error and data not missing at random. The famous Hausman (1978) specification test that compares fixed-effect estimates (e.g., from CML estimation) with random-effects estimates (e.g., from MML estimation) is routinely used in the context of model (3). Contrary to common belief, a significant test cannot be interpreted as flagging unobserved confounding because cluster-level endogeneity can arise for a variety of reasons.

In psychology, split-plot analysis of variance (ANOVA) is sometimes used for repeated measures designs. The hypothesis test for a within-subject effect is in this case robust against cluster-level endogeneity. However, the focus is traditionally solely on hypothesis testing and not estimation of parameters or effect sizes. A very similar fixed-effects approach known as difference-in-differences in economics is popular for estimating effects in natural/quasi experiments (e.g., Angrist & Pischke, 2009).

Hybrid estimation approaches can be obtained by combining CML with other estimation methods:

- CML and MML (and related) estimation: In a Rasch context, Andersen and Madsen (1977) used CML to estimate item parameters ${\varvec{\beta }}$ and subsequently MML to estimate the expectation and variance of a parametric person distribution, given ${\widehat{{\varvec{\beta }}}}^{\scriptscriptstyle \mathrm CML}$. For a linear mixed model with random slopes, Verbeke et al. (2001) used a conditional likelihood to eliminate the cluster-specific intercept $\zeta _j$ (and ${\varvec{\gamma }}$), and MML or restricted maximum likelihood (REML) to estimate ${\varvec{\beta }}$, $\sigma ^2$ and the covariance matrix of the slopes in ${\varvec{\zeta }}_j$. Tibaldi et al. (2007) combined CML and composite-likelihood estimation of crossed random-effects models with identity and logit links.
- CML and instrumental variables (IV) estimation: For model (3) with identity link, Hausman and Taylor (1981) proposed a multi-stage estimation approach where CML is used to estimate ${\varvec{\beta }}$ (and $\sigma ^2$) followed by IV estimation of ${\varvec{\gamma }}$ (and the variance of $\zeta _j$) for given ${\widehat{{\varvec{\beta }}}}^{\scriptscriptstyle \mathrm CML}$. The instruments are internal in the sense that they are constructed from the covariates ${\mathbf {x}}_{ij}$ and ${{\mathbf {v}}}_j$ in the model. All parameters can be consistently estimated if one can correctly designate which unit- and cluster-specific covariates are cluster-level endogenous. In a panel data setting with identity link, Lee (2002) used first differencing to remove cluster-specific intercepts. Subsequently, he used IV methods to estimate ${\varvec{\beta }}$, where various exogeneity assumptions for the $\epsilon _{ij}$ dictate if covariates at an occasion can serve as internal instruments for covariates at other occasions.
- CML and Bayes estimation: Conditional likelihoods have been used in conjunction with prior distributions for model parameters in Bayesian inference. This was motivated by Diggle et al. (2000) to handle retrospective sampling and by Lancaster (2004) to handle unobserved confounding. It is worth pointing out that standard Bayesian inference that ignores cluster-level endogeneity performs similarly to standard MML estimation.
- Mixed-type ML estimation: Cook and Farewell (1999) discussed the construction of mixed-type likelihoods where the likelihood contributions are of different types for different clusters. They considered a version of linear predictor (3) with a logit link, where conditional likelihoods were used for small clusters whereas joint likelihoods were used for large clusters.

An interesting recent development is the use of conditional likelihoods in double-robust estimation. Zetterquist et al. (2019) consider model (3) with a logit link and a binary treatment $x_{ij}$ of interest. They argue that consistency for the corresponding $\beta$ can be achieved if at least one of the following approaches is consistent for $\beta$: i) CML estimation of $\beta$ in the prospective model (3) for $y_{ij}$ or ii) CML estimation of $\beta$ as the coefficient of $y_{ij}$ in a retrospective logit model for $x_{ij}$ with a cluster-specific intercept. One need not know which of the models is correct so there are in this sense two opportunities of getting it right.

In closing, it is evident that we have drawn on and extended results not just from psychometrics but also from other “ics”, such as econometrics, biometrics and statistics, in this address. Unfortunately, progress in psychometrics has been hampered by a paucity of cross-fertilization from the other “ics” — but the opposite is certainly also true! A case in point is the extensive literature on covariate measurement error where the psychometric wheel is regularly reinvented, albeit often in a somewhat square fashion. We end with a perceptive citation from a *Psychometrika* article by the renowned econometrician Arthur Goldberger (Goldberger, 1971, p. 83):

“Economists and psychologists have been developing their statistical techniques quite independently for many years. From time to time, a hardy soul strays across the frontier but is not met with cheers when he returns home.”
